# Frequency-dependent topological polaritons in carbon nanotube array/hBN heterostructures

**DOI:** 10.1038/s41467-026-71100-z

**Published:** 2026-03-24

**Authors:** Yufeng Xie, Kaijun Feng, Zhichun Zhang, Saiqun Ma, Zhenghan Wu, Yi Chen, Chengjia Zhang, Liguo Wang, Kenji Watanabe, Takashi Taniguchi, Qi Liang, Xiangdong Guo, Qing Dai, Zhiwen Shi

**Affiliations:** 1 State Key Laboratory of Micro-nano Engineering Science, Key Laboratory of Artificial Structures and Quantum Control (Ministry of Education), School of Physics and Astronomy and Tsung-Dao Lee Institute, Shanghai Jiao Tong University, Shanghai, China; 2 Institute of Information Functional Materials, Shanghai Key Laboratory of Atomic-Level Intelligent Manufacturing of Materials and Devices, School of Materials Science and Engineering, Shanghai Jiao Tong University, Shanghai, China; 3 Research Center for Electronic and Optical Materials, National Institute for Materials Science, Tsukuba, Japan; 4 Research Center for Materials Nanoarchitectonics, National Institute for Materials Science, Tsukuba, Japan; 5 Collaborative Innovation Centre of Advanced Microstructures, Nanjing University, Nanjing, China

**Keywords:** Carbon nanotubes and fullerenes, Nanophotonics and plasmonics, Nanophotonics and plasmonics, Carbon nanotubes and fullerenes, Nanophotonics and plasmonics

## Abstract

Plasmons in carbon nanotubes (CNTs) have attracted significant attention due to their strong spatial confinement and high-quality factors. However, constrained by CNTs’ intrinsic electronic characteristics, modulation of their plasmon dispersion relations remains a significant challenge. Here, we report frequency-dependent topological polaritons arising from the coupling between hyperbolic plasmons and phonon polaritons in CNT-array/hexagonal boron nitride (hBN) heterostructures. In particular, we achieved a controllable topological transition of the polariton wavefront from hyperbolic to elliptical. Moreover, we demonstrated a whispering-gallery polaritonic mode confined in closed-loop CNT array on hBN. Our findings provide fundamental insights into optical topological transitions in low-dimensional heterostructures, and a promising route to manipulate light propagation and energy transfer at the nanoscale.

## Abstract (web summary)

Plasmons in carbon nanotubes (CNTs) benefit from high quality factors but the modulation of their dispersion relation remains challenging. Here the authors report the observation of hybrid plasmon-phonon polaritons in CNT arrays coupled with 2D hexagonal boron nitride, showing frequency-dependent topological transitions and whispering-gallery modes.

## Introduction

Polaritons, hybrid quasiparticles resulting from the coupling between collective oscillations of particles in materials and electromagnetic waves, have demonstrated remarkable potential of nanophotonic engineering for manipulating light-matter interactions^1–3^. Notably, in hyperbolic media, such as hexagonal boron nitride (hBN)^4–9^ and α-phase molybdenum trioxide (α-MoO_3_)^10–18^, phonon polaritons exhibit pronounced directional anisotropy, with their isofrequency curves and propagation wavefronts forming distinct hyperbola-like shapes. Furthermore, control over the propagation direction of these hyperbolic polaritons through layer stacking and twist angles has been widely reported^19–26^. Yet, the limited spectral range of these phonon polaritons has significantly hindered their practical applications. Plasmon polaritons^27,28^, particularly in one-dimensional carbon nanotubes (CNTs)^29–35^, present exceptional properties, including deep subwavelength confinement and broad spectral range. Recently, closely packed homochiral CNT arrays have been successfully fabricated^36^, and signatures of hyperbolic Luttinger-liquid plasmons have been reported in this distinct material structure^37^. However, despite their extraordinary properties, control of the dispersion relation or isofrequency contour of the plasmons in CNT arrays has so far remained unexplored yet.

Here, with a homemade scattering-type scanning near-field optical microscope (s-SNOM), we experimentally demonstrate nanoscale frequency-dependent topological polaritons—specifically, the topological transition of polariton iso-frequency contours between open and closed-loop shapes, in CNT-array/hBN heterostructures. Our results achieved control over the polariton dispersion, topology, and propagation by simply tuning the excitation frequency. The control is achieved through strong coupling between anisotropic Luttinger-liquid plasmons in close-packed identical CNT arrays and in-plane isotropic phonon polaritons in hBN when the excitation frequency lies within the Reststrahlen band of hBN substrate, leading to a topological transition of plasmon isofrequency contour that evolves from hyperbolic to elliptical. Furthermore, using closed-loop CNT arrays as circular nanoscale resonators and leveraging the high-quality factor of the coupled mode, we demonstrated polaritonic whispering-gallery modes in these closed-loop CNT arrays. Our study offers an alternative avenue to control nanoscale light through strong light-matter interaction, and a paradigm for applications in compact nanophotonic devices.

## Results

### Material structure, measurement scheme, and working principle

**Fig. 1:**
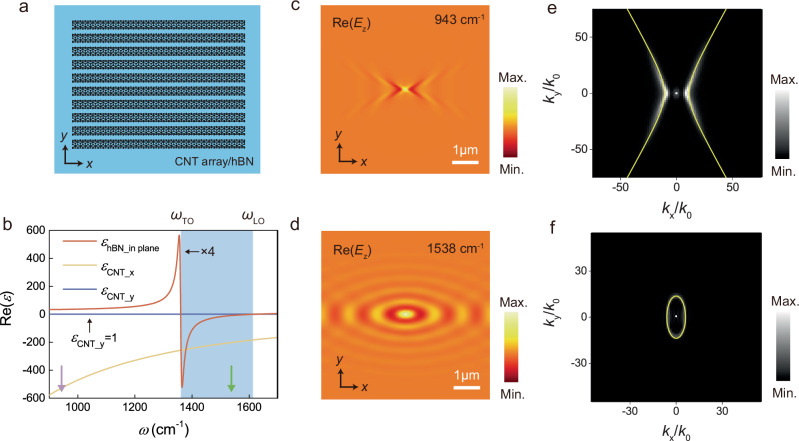
Numerical simulations of polaritons with different topologies in a carbon-nanotube (CNT) array/hexagonal-boron-nitride (hBN) heterostructure. **a** A schematic of the structure of a close-packed, parallel CNT array on an hBN substrate. **b** The dielectric functions of hBN and the CNT arrays, with the blue area marking the Reststrahlen band of hBN. The *ε*_hBN_in plane_ refers to the in-plane dielectric function of hBN. The *ε*_CNT_x_ and *ε*_CNT_y_ refer to the x-directional and y-directional dielectric functions of CNT array, respectively. The “x4” label means that the in-plane permittivity of hBN has been multiplied by four so that the dielectric functions of both hBN and CNTs can be displayed together on the same *y*-axis. The purple and green arrows refer to the excitation frequencies corresponding to the simulated distributions in (c) and (d), respectively. ${\omega }_{{{{\rm{TO}}}}}$ and ${\omega }_{{{{\rm{LO}}}}}$ refer to the transverse and longitudinal optical phonon frequencies of hBN, respectively. Simulated spatial distribution of the real part of the polariton field Re(*E*_z_) at frequencies of 943 cm^−1^ (**c**) and 1538 cm^-1^ (**d**), respectively, simulated just above the CNT-array/hBN. Fourier transforms of the polariton fields shown in (**c**, **d**), revealing hyperbolic (**e**) and elliptical (**f**) isofrequency curves, respectively. The *k*_x_ and *k*_y_ refer to the x-directional and y-directional momentum, while the *k*_0_ refers to the photon momentum in free space.

Our material structure composed of a close-packed array of identical CNTs on hBN substrate, a schematic of which is depicted in Fig. 1a. To quantitively analyze polariton modes within the CNT-array/hBN heterostructure, we carried out numerical simulations of the eigen electromagnetic modes in the structure using a finite element method (COMSOL Multiphysics). The structure is modeled as a dielectric medium composed of an in-plane strongly anisotropic layer^38–40^—representing the CNT array, and an in-plane isotropic layer—representing the hBN flake. The specific dielectric function used in our simulation can be found in Fig. 1b (see more details in Supplementary Note 2). An electrical dipole is placed at the center of the plane and serves as the excitation source. Numerically simulated field distributions of hybrid polariton modes at two representative excitation frequencies (943 cm^-1^ and 1538 cm^−1^) are shown in Fig.1c, d, respectively. At excitation frequency of 943 cm^-1^, the wavefront of the polariton mode exhibits hyperbolic. The hyperbolic plasmon originates from the strongly anisotropic conductivities of the layer of CNT array between the axial direction (x-direction) and the radial direction (y-direction). At excitation of 1538 cm^−1^, the wavefront becomes elliptical. The topological change of the wavefront is a result of the dramatic change in the dielectric function of the hBN layer.

It has been widely known that the two Reststrahlen bands (type I and II) of hBN support the propagation of phonon polaritons^4,7,8^. The type I band with out-of-plane dielectric function ${\varepsilon }_{{{{\rm{z}}}}} < 0$ and in-plane dielectric function ${\varepsilon }_{{{{\rm{t}}}}} > 0$, extends over the frequency range $\omega=760 \sim 825\,{{{{\rm{cm}}}}}^{-1}$, and the type II band with ${\varepsilon }_{{{{\rm{z}}}}} > 0\,{{{\rm{and}}}}\,{\varepsilon }_{{{{\rm{t}}}}} < 0$, spans the range $\omega=1360 \sim 1614\,{{{{\rm{cm}}}}}^{-1}.$ In this study, we focus exclusively on the analysis of polaritons at frequency range around the type II band. When the frequency is outside the type II Reststrahlen band, hBN exhibits positive in-plane permittivity (depicted in Fig. 1b), and no phonon polaritons are supported. As a consequence, the wavefront of plasmons in the CNT array is hyperbolic, representing the intrinsic plasmon mode of the CNT array. Once the frequency (e.g., 1538 cm^−1^) falls into the type II Reststrahlen band, a topological transition occurs, and the wavefront transforms into an elliptical shape, owing to the negative isotropic in-plane permittivity of hBN. Noted that the frequency at which this topological transition occurs is conditioned to the appearance of well-defined phonon polaritons in hBN. The strong coupling between hyperbolic plasmons in CNT array and isotropic in-plane phonon polaritons in hBN results in the elliptical-shaped wavefront. Figure 1e, f illustrate the corresponding Fourier transform of Fig. 1c, d, where the yellow lines represent the fitted hyperbolic and elliptical isofrequency contour, respectively.

**Fig. 2:**
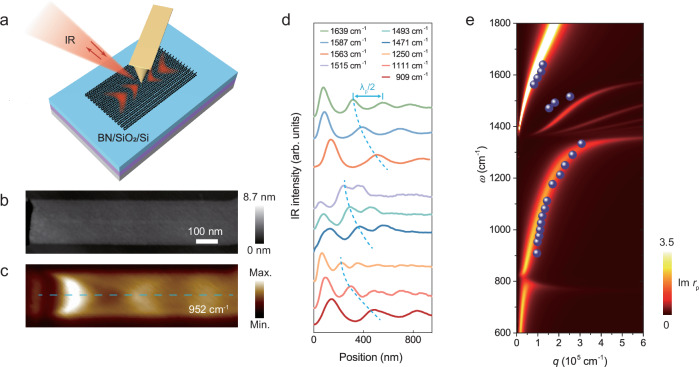
Observation of the hybrid modes between plasmons and phonon polaritons in CNT-array/hBN heterostructure. **a** Schematic of infrared (IR) nano-imaging of polaritons in a CNT-array/hBN using scattering-type scanning near-field optical microscope (s-SNOM). **b** Atomic force microscope (AFM) topography image showing the CNT array on hBN substrate. **c** Infrared nano-imaging of hybrid polaritons in the CNT-array/hBN at a frequency of 952 cm^−1^. The extracted quantity represents the magnitude of the electric field. **d** Near-field profiles of the sections marked by the dashed line in (**c**). The dashed guide lines show the trend of the interference peaks with frequency. *λ*_p_ refers to the plasmon wavelength. **e** Comparison of calculated and experimental dispersion relations of hybrid polaritons in the CNT-array/hBN. Experimental data points are shown as dots, while the calculated dispersion is depicted by a false-color map of the imaginary part of the reflection coefficient.

To experimentally demonstrate the inter-layer polariton coupling and the topological transition, we grew close-packed CNT arrays on hBN substrate using chemical vapor deposition (CVD)^36,41,42^; further details are provided in the *Method* section. Notably, the CNTs within each array exhibit uniform chirality and a close-packed arrangement, allowing the CNT array to be effectively described and modeled as an effective anisotropic medium^36^ (see more details in Supplementary Note 1). Infrared nano-imaging of the polariton response in the CNT array is achieved through a home-made s-SNOM, as illustrated in Fig. 2a. A gold-coated atomic force microscope (AFM) tip, illuminated by an infrared beam, serves as an exciter to launch polaritons in the CNT arrays. The excited polaritons propagate along the surface and reflect at the ends of the CNT array. The reflected polariton waves alter the local electromagnetic field beneath the tip apex, which is then scattered into the far field and detected by a mercury cadmium telluride (MCT) detector. In order to better capture the polariton interference pattern, an additional AFM cutting process^43^ is typically applied to the CNT array to create a sharp terminal boundary that reflects polariton waves with a well-defined phase (see *Methods* for more details).

### Coupling between CNT plasmons and hBN phonon polaritons

To effectively visualize the polaritons within the CNT arrays, it is experimentally necessary to reduce the intensity of the hBN phonon polaritons to match that of the CNT plasmons. Therefore, we selected a relatively thin hBN flake, measuring 55 nm in thickness, to demonstrate the polaritonic coupling. Figure 2c shows the near-field optical image of the CNT-array/hBN heterostructure at excitation frequency of 952 cm^−1^, while Fig. 2b presents the corresponding topography image. Notably, the polaritons exhibit a hyperbolic wavefront, a result of the intrinsic hyperbolic plasmons in the CNT array. The polariton oscillation profiles along the *x*-axis (the tubes’ axial direction) at various frequencies are plotted in Fig. 2d, revealing a clear change in the oscillation period. It should be noted that in our reflection imaging scheme, the polariton oscillation period corresponds to half the polariton wavelength. As the frequency decreases, the polariton wavelength generally increases, but exhibits two abrupt decreases at specific frequencies, dividing the behavior into three distinct regimes. This unusual behavior presumably results from the coupling between CNT array plasmons and the hBN phonon polaritons.

The near-field infrared response of the CNT-array/hBN system is vividly illustrated by the calculated false color map of the imaginary part of the reflectivity (Fig. 2e), highlighting the frequency ($\omega$)-momentum (${k}_{{{{\rm{x}}}}}$) dispersion relation of the eigen polariton modes. In contrast to isotropic materials, the hyperbolic hBN supports multiple distinct branches of phonon polaritons between its ${\omega }_{{{{\rm{TO}}}}}$ and ${\omega }_{{{{\rm{LO}}}}}$ frequency range, corresponding to quantized waveguide modes oscillating between the top and bottom surfaces of the hBN slab, as detailed in Supplementary Note 3. As a result, the coupled modes of hybrid polaritons exhibit multiple branches within the frequency range. The CNT array plasmons induce significant modifications to the coupled modes, causing the hybrid modes to display a clear blue shift relative to the original phonon polariton frequencies (see the hBN phonon polariton dispersion relations in Supplementary Fig. 2). The experimental results, represented by blue dots extracted from the polariton oscillation profiles, are distributed across three distinct bands and show excellent agreement with the theoretical calculations. The second experimental band fits with mode with the second-lowest momentum within the Reststrahlen band. The discrepancy between the experimental data and the calculated dispersion in the Reststrahlen band arises because our measurement captures a superposition of the fundamental and second-order modes of the hybrid polariton. Besides, the oscillation profiles of this second band (the three middle curves in Fig. 2d) seem to originate from multiple momentum components, corresponding to several sub-branches within the Reststrahlen band. These multiple sub-branches arise from various hBN phonon-polariton modes^4,44^. Moreover, we calculated the hybrid polariton dispersion using the finite element method and obtained consistent results (Supplementary Note 4). All these findings closely correspond with the dispersion characteristics of graphene/hBN plasmon-phonon polaritons reported in previous studies^45^, demonstrating subtle but noticeable differences.

### Coupling induced topological transition in polariton

**Fig. 3:**
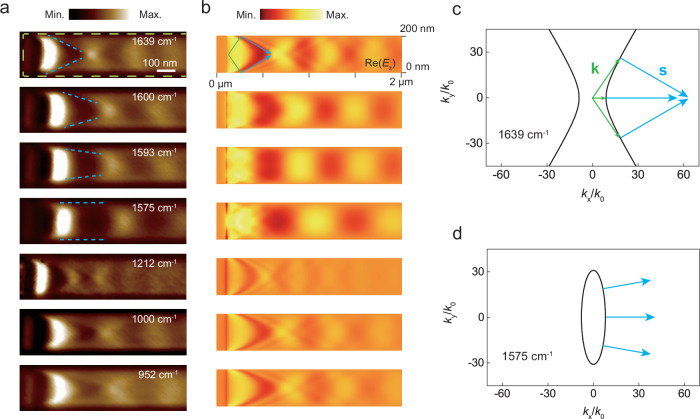
The topological transition of hybrid polaritons in the CNT-array/hBN heterostructure revealed by nano-imaging. **a** Infrared nano-imaging of polaritons in the CNT-array/hBN at different frequencies. The extracted quantity represents the magnitude of the electric field. The blue dashed lines serve as guides to illustrate the convergent or divergent trends of the polaritons. The yellow dashed lines mark the boundary of the CNT array. **b** Numerically simulated polariton fields Re(*E*_z_) in the CNT-array/hBN corresponding to the frequencies shown in (**a**). **c** Isofrequency contour of hyperbolic polaritons at 1639 cm^−1^. Green arrows represent wavevectors, while blue arrows represent Poynting vectors. **d** Isofrequency contour of elliptical polaritons at 1575 cm^−1^.

We then carried out an in-depth investigation into this coupled polariton mode, which led to the observation of an intriguing topological wavefront transition. The detailed, step-by-step process of this transition is thoroughly illustrated in Fig. 3a. Remarkably, as the frequency decreases and enters the Reststrahlen band, coupling with hBN polaritons causes the wavefront of polaritons to undergo a clear transition from convergent to divergent propagation. Consequently, the interference pattern changes distinctly from a hyperbolic to a rectangular (or parallel) geometry, signaling a topological transition. Figure 3c, d show the isofrequency contours of the hybrid polaritons at 1639 cm^−1^ and 1575 cm^−1^, corresponding to the hyperbolic and elliptical curves, respectively. The green and blue arrows indicate the wavevectors and Poynting vectors. Importantly, because the hyperbolic plasmon isofrequency curve is open, energy flow tends to converge, whereas in the elliptical case it diverges. Our experimental results suggest a characteristic transition of polaritonic wavefront from hyperbolic to elliptical, which reverts back to hyperbolic upon exiting the Reststrahlen band. Numerical simulations supporting these findings are presented in Fig. 3b and show excellent agreement with the experiments. Additionally, Supplementary Fig. 6 illustrates the progression of the calculated isofrequency contours for the hybrid polaritons at various frequencies, demonstrating the complete transition of the polariton topology from elliptical to hyperbolic.

To further confirm this topological transition, we fabricated a hole in the CNT arrays using AFM etching to serve as a point reflector for polaritons. The tip-launched polaritons reflect off this hole and produce interference patterns, yielding standard near-field images of hyperbolic and elliptical polaritons, as depicted in Supplementary Fig. 4. The top image shows the hyperbolic plasmon wavefront at an excitation frequency of 943 cm^−1^, while the bottom image shows the elliptical wavefront coupled with hBN phonon polaritons at 1563 cm^−1^. It is important to note that hyperbolic plasmons in CNT arrays originate from their ability to propagate in the *x*-axis (the axial direction) but not the y-axis. Thanks to the well-defined phonon polaritons along the tube’s radial direction, when the in-plane permittivity of hBN turns negative, hybrid polariton propagation is enabled even in the previously restricted direction, resulting in an elliptical wavefront.

To clarify this transition further, we performed numerical simulations of polaritons on CNT arrays of varying widths. Since our CNT arrays are limited in size along the tube radial direction, we demonstrate the polariton patterns in arrays with different numbers of CNTs, as depicted in Supplementary Fig. 5. Polaritons were excited at the array boundary (marked by a dashing line) by an incident plane electromagnetic wave polarized along the nanotubes. When the array is sufficiently wide, the polariton wavefronts become obviously hyperbolic and elliptical, at excitation frequencies of 952 cm^−1^ and 1575 cm^−1^, respectively. With a reduction in the array width, the polariton pattern gradually changes and aligns more closely with our experimental images, which further confirms the polariton wavefronts in Fig. 3a are hyperbolic at 952 cm^−1^ and 1639 cm^−1^, while elliptical at 1575 cm^−1^.

### Observation of a tunable whispering-gallery polaritons

**Fig. 4:**
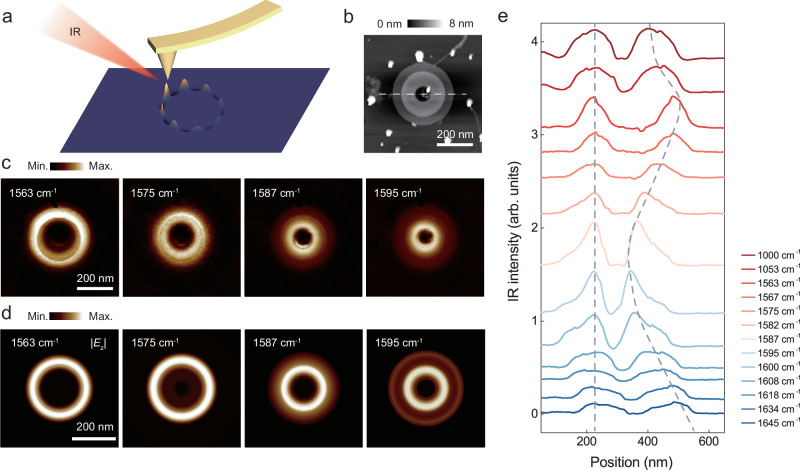
Whispering-gallery mode of hybrid polaritons in a closed-loop CNT array ring on hBN substrate. **a** Schematic of infrared nano-imaging of whispering-gallery polaritonic mode in the CNT-array/hBN by using s-SNOM. **b** AFM topography image of a closed-loop CNT array ring on hBN. **c** Infrared nano-imaging of polaritons in the closed-loop CNT-array on hBN at different frequencies. The extracted quantity represents the magnitude of the electric field. **d** Numerically simulated polariton fields $\left|{E}_{z}\right|$ in the closed-loop CNT array on hBN. **e** Near-field line profiles along the sections marked by the dashed line in (**b**). The gray dashed lines denote the frequency variation of the two interference enhancement peak positions.

Next, we demonstrate a tunable whispering-gallery mode within the CNT-array/hBN heterostructure. Due to the absence of electronic Ohmic loss^44,46,47^, hBN phonon polaritons are characterized by exceptionally long lifetimes and high-quality factors, thereby enhancing the propagation length of the hybrid polaritons in the CNT arrays. On the other hand, according to the calculated dispersion results (Fig. 2e), multiple distinct branches of hybrid polaritons exist in the CNT-array/hBN heterostructure, which leads to the polariton wavelength changes significantly with the excitation frequency. This, in turn, facilitates the design of polaritonic resonators. To test this idea, we fabricated closed-loop CNT arrays on hBN substrate. This distinct structure was created by repeatedly winding a nanotube in a spiral pattern resembling a mosquito coil, ultimately forming a circular, donut-like architecture, as illustrated in Fig. 4b and Supplementary Fig. 8.

We then performed infrared nano-imaging on these closed-loop structures and successfully observed polaritonic whispering-gallery modes (Fig. 4a). The AFM topography of the CNT array is shown in Fig. 4b, while Fig. 4c and Supplementary Fig. 7 are near-field optical images with varying excitation frequencies. Remarkably, near the frequency ${\omega }_{{LO}}$, the polariton interference pattern in the CNT array changed sharply with frequency.

At a frequency of 1563 cm^−1^, the inner ring exhibited almost no near-field optical response (dark), whereas the outer ring displayed strong response (bright). This phenomenon is presumably caused by destructive interference in the inner ring and constructive interference in the outer ring, as the closed-loop array can be divided into several sub-rings with different perimeters. Based on the polariton dispersion relations, we estimated that the perimeter of the outer tubes is approximately the wavelength of the polaritons, while that of the inner tubes is roughly half the wavelength. Notably, as frequency increasing, the constructive interference ring first contracted and then expanded. To clearly illustrate this behavior, we extracted and plotted the polariton line profiles along the dashed line marked in Fig. 4b. These profiles reveal that the constructive polariton interference peaks vary significantly with the excitation frequency, as shown in Fig. 4d. By aligning one of these peaks, we provided a more intuitive comparison of the peak-to-peak distance and intensity variations between the two peaks. Firstly, the distances between the two peaks exhibited clear oscillations with frequency, demonstrating the resonance of polariton waves in nanotube rings of varying diameters. Secondly, as the two interference peaks move closer together, the interference pattern becomes more strongly constructive (as shown in Fig. 4e). This phenomenon can be attributed to the fact that smaller peak spacing corresponds to shorter propagation lengths, which are typically associated with lower energy losses, leading to the more strongly constructive interference. Another CNT ring exhibiting similar polaritonic resonant behavior can be found in Supplementary Note 9, and further evidence for whispering-gallery modes is provided in Supplementary Note 11.

The realization of these remarkable features stems from the unique physical properties of the materials and the precisely controlled geometric design of the nano-ring structures. Specifically, within the closed-loop arrays of natural CNT crystals, the synergy of the low-loss characteristics of the Luttinger liquid electronic state, uniform chirality, and near-atomic-level smooth, low-loss ring-shaped interfaces enables efficient optical field confinement and the formation of strong optical resonances (see more details in Supplementary Note 10). These properties create ideal conditions for the generation and high-fidelity propagation of whispering-gallery modes. This on-chip natural whispering-gallery nanocavity structure features a diameter reduced to approximately one hundred nanometers. Compared to traditional low-dimensional polaritonic whispering-gallery nanocavities^48^, its characteristic dimensions have improved by nearly an order of magnitude. Its mode volume is as small as on the order of 10^−6^. The high compression ratio and quality factor not only preserve optical state coherence, but also provide robust support for exploring strong light–matter interactions. Furthermore, by adjusting geometric parameters, the resonant frequency and mode distribution of the whispering-gallery nanocavity can be precisely tuned, providing a promising pathway for achieving integrated optoelectronic systems. Consequently, single-chirality CNT-based whispering-gallery nanocavities advance our understanding of light–matter interactions and showcase the unique benefits of Luttinger liquid plasmons in low-loss optics, offering vital theoretical and experimental support for high-performance integrated optoelectronics.

## Discussion

In summary, we utilized the atomically smooth interfaces of close-packed, high-aligned, and homochiral CNT arrays to achieve strong coupling between Luttinger liquid electronic hyperbolic plasmons and phonon polaritons in hBN. This coupling unveiled the topological transition of polaritonic wavefronts from hyperbolic to elliptical shapes. The ability to tune hyperbolic plasmons across a broad frequency range provides a fresh perspective on nanoscale topological transitions. Moreover, leveraging the high compression ratio and low-loss characteristics of these hybrid polaritons, we directly observed polaritonic whispering-gallery modes within closed-loop CNT arrays with diameter reduced to about a hundred nanometers, exhibiting capabilities for on-chip manipulation of whispering-gallery optical fields. Therefore, our work not only introduces a distinct topological hybrid polariton material system but also unlocks further opportunities for the realization of extreme on-chip whispering-gallery nanocavities, establishing a strong foundation for the development of integrated nanoscale optoelectronic devices.

## Methods

### Growth of CNT arrays

The hBN flakes were initially mechanically exfoliated onto SiO_2_/Si substrates. To ensure surface cleanliness, the samples were subsequently exposed to hydrogen plasma at 300 °C, effectively removing all organic residues. Following this pretreatment, a precisely controlled 0.05 nm-thick iron (Fe) film was deposited onto the hBN-coated SiO_2_/Si surfaces using thermal evaporation, which served as catalytic nanoparticles for subsequent CNT arrays growth. The prepared substrates were then transferred into a quartz tube furnace (Anhui BEQ Equipment Technology) and subjected to a controlled heating process under a mixed atmosphere of hydrogen and argon at atmospheric pressure, gradually elevating the temperature to the optimal growth temperature of ~850 °C^41,42^. Upon reaching this temperature, the argon gas flow was replaced with methane to initiate the CNT arrays growth process. After a standard growth period of 30 min, the system was systematically cooled down to room temperature while maintaining a protective atmosphere of hydrogen and argon.

### AFM cutting of CNT arrays

The cutting of CNT arrays for designed structures is performed in the atmosphere using a standard atomic force microscope (AFM). First, AFM is used to locate the CNT arrays grown on hBN/SiO_2_/Si substrate. Then, a 50 kHz AC voltage with an amplitude of 10 V is applied between the conductive AFM tip and the Si layer of the substrate. The tip scans very slowly across the CNT array in tapping mode with a lift-down distance of 90–120 nm. Local anodic oxidation only occurs for CNTs underneath the tip with the assistance of a water bridge. Therefore, the array along the tip’s path is etched to be a smooth artificial boundary. The point hole is etched in the same way.

### Infrared nano-imaging

For near-field infrared nano-imaging, we employed a custom-built scattering-type scanning near-field optical microscope (s-SNOM) that integrates a tapping-mode atomic force microscope (AFM, Bruker Innova) with a CO_2_ laser and a Daylight Solution quantum cascade laser (QCL). The system operates by focusing a mid-infrared wavelength laser (900–1650 cm^−1^) beam onto the apex of a conductive AFM probe, generating a highly localized enhanced optical field. This near-field interacts with the sample, and the resulting scattered light, which encodes local optical information, is collected in the far field using a mercury cadmium telluride (MCT) detector (KLD-0.1-J1, Kolmar). To suppress background scattering, the collected signal is demodulated using a lock-in amplifier (Zurich Instruments HF2LI). This configuration allows for the simultaneous acquisition of high-resolution near-field optical images alongside topography information during measurements.

### Numerical simulations

COMSOL Multiphysics is used to simulate the polariton patterns in CNT-array/hBN heterostructure. CNT array is modeled by a plane with the same size of experimental samples. We estimated the conductivity of CNTs utilizing the method reported by previous literature and selected an appropriate value as the complex permittivity^49^. For the simulations shown in Fig. 1c, d, a point electric dipole was placed at the center of CNT arrays to excite polaritons at a varying oscillating frequency. For the simulations shown in Fig. 3b, a beam of light polarized perpendicularly to the vertical boundary was irradiated onto the plane surface to excite polaritons at a varying oscillating frequency. The electric field component perpendicular to the plane (*E*_z_) was recorded over the plane surface.

### Dispersion calculations

The calculations of the dispersion shown in Fig. 2e and Supplementary Fig. 2 were performed using a generalized 4 × 4 transfer matrix formalism^50^. In short, by incorporating the permittivity of CNT arrays and hBN, the solution of reflectivity, ${r}_{p}(q,\omega )$, can be determined. Polariton mode corresponds to the frequencies at which the imaginary parts of ${r}_{p}(q,\omega )$ reach the maximum. For each frequency, the corresponding polariton wavevector can be extracted.

## Supplementary information

Supplementary Information

Transparent Peer Review file

## Data Availability

Relevant data supporting the key findings of this study are available within the article and the Supplementary Information file. All raw data generated during the current study are available from the corresponding authors upon request.
