# Supplementary material for: Frequency-dependent topological polaritons in carbon nanotube array/hBN heterostructures
Source: Nat Commun. 2026 Mar 24;17:5181. doi: 10.1038/s41467-026-71100-z (PMC13254115; doi:10.1038/s41467-026-71100-z)
Supplement: Supplementary file 1 — Supplementary Information [file 41467_2026_71100_MOESM1_ESM.pdf]

Supplementary Information for

# Frequency-dependent topological polaritons in carbon nanotube array/hBN heterostructures

Yufeng Xie[1], Kaijun Feng[2], Zhichun Zhang[1], Saiqun Ma[1], Zhenghan Wu[1], Yi Chen[1], Chengjia Zhang[1], Liguo Wang[1], Kenji Watanabe[3], Takashi Taniguchi[4], Qi Liang[1], Xiangdong Guo[2]*, Qing Dai[2]*, Zhiwen Shi[1,5]*

[1]State Key Laboratory of Micro-nano Engineering Science, Key Laboratory of Artificial Structures and Quantum Control (Ministry of Education), School of Physics and Astronomy and Tsung-Dao Lee Institute, Shanghai Jiao Tong University, Shanghai 200240, China

[2]Institute of Information Functional Materials, Shanghai Key Laboratory of Atomic-Level Intelligent Manufacturing of Materials and Devices, School of Materials Science and Engineering, Shanghai Jiao Tong University, Shanghai 200240, China

[3]Research Center for Electronic and Optical Materials, National Institute for Materials Science, 1-1 Namiki, Tsukuba 305-0044, Japan.

[4]Research Center for Materials Nanoarchitectonics, National Institute for Materials Science, 1-1 Namiki, Tsukuba 305-0044, Japan.

[5]Collaborative Innovation Centre of Advanced Microstructures, Nanjing University, Nanjing 210093, China.

*To whom correspondence should be addressed: guoxiangdong@sjtu.edu.cn, daiqing@sjtu.edu.cn, zwshi@sjtu.edu.cn

**Supplementary Note 1. AFM characterizations of carbon nanotube arrays**

**Supplementary Note 2. Estimation of the permittivity of carbon nanotube arrays**

**Supplementary Note 3. Calculated dispersion relation of CNT array plasmons, hBN phonon polaritons, and the hybrid mode**

**Supplementary Note 4. Numerical simulations of dispersion relation of hybrid polaritons**

**Supplementary Note 5. IR nano-imaging of topological transition of polaritons reflected by a point defect of a hole**

**Supplementary Note 6. Numerical simulations of polaritons in CNT-array/hBN of different widths**

**Supplementary Note 7. The continuous evolution of iso-frequency contours across the transition region**

**Supplementary Note 8. The evolution of the whispering-gallery mode of the hybrid polaritons with frequency**

**Supplementary Note 9. An additional sample showing the whispering-gallery mode of hybrid polaritons**

**Supplementary Note 10. The quality factor of hybrid polaritons in closed-loop CNT array ring on hBN**

**Supplementary Note 11. The evolution of the hybrid polaritons in a larger closed-loop CNT array ring on hBN**

## Supplementary Note 1. AFM characterizations of carbon nanotube arrays

Here, we performed a detailed atomic force microscopic (AFM) characterization of an as-grown CNT array, as depicted in Supplementary Figure 1. The AFM topography image in Supplementary Figure 1a displays a representative CNT array, and a zoomed-in AFM image in Supplementary Figure 1b was obtained using super-sharp AFM probes. This method has been reported in our previous literature[1]. Parallel CNTs within the array were well aligned (with a constant intertube distance of 0.33 nm). A line profile in Supplementary Figure 1c shows a uniform period of 1.62 nm across the CNTs, indicating the same diameter.

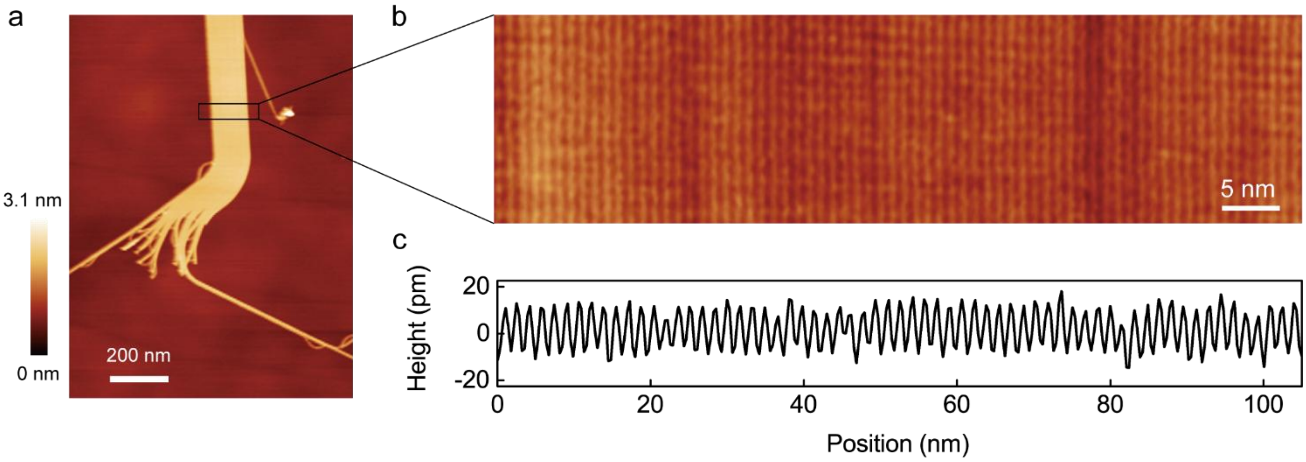


**Supplementary Figure 1. AFM characterization of a representative CNT array with uniform chirality. a**, AFM topography of a CNT array on hBN. **b**, A zoom-in image of the CNT array (indicated by the square area in (a)), showcasing close-packed individual CNTs. **c**, Cross-sectional line-profile of the array in (b).

## Supplementary Note 2. Estimation of the permittivity of carbon nanotube arrays

The conductivity of CNT arrays can be obtained by following previously reported literatures[2, 3], and similar derivations have been reported in our previous literature[4]. First, the axial complex surface conductivity of an individual metallic zigzag CNT with chirality (m, 0) at frequencies below the first inter-band transition is:

$$\sigma_{CNT}(\omega) \cong i\frac{2\sqrt{3}e^2\gamma_0}{\mathrm{m}\pi\hbar^2(\omega+i\nu)} \tag{1}$$

where $e$ is the electron charge, $\gamma_0 = 2.7eV$ is the overlapping integral, $\nu = 3.33 \times 10^{13}\ s^{-1}$ is the relaxation frequency, and $\hbar$ is the reduced Plank's constant.

In numerical simulations, surface conductivity needs to be transformed into bulk conductivity. We then consider an air-filled CNT as a nanowire (NW) and convert its conductivity to that of the NW. According to the distributed Ohm's law, the electrical field over the cross-section is:

$$I(z,\omega) = \frac{E_z(z,\omega)}{Z(z,\omega)} \tag{2}$$

where $I(z,\omega)$ is the net current across the CNT cross-section at z, $E_z(z,\omega)$ is the electrical field component along z in the CNT, and $Z(z,\omega)$ is the distributed impedance. For a NW, we have

$$Z_{NW,bulk}(z,\omega) = Z_{CNT,surf}(z,\omega) \tag{3}$$

Then we have,

$$\iint_{cross\ section} \sigma_{NW}(z,\omega)dA = \int_{perimeter} \sigma_{CNT}(z,\omega)dl \tag{4}$$

where $\sigma_{NW}(z,\omega)$ is the bulk conductivity of the NW and $\sigma_{CNT}(z,\omega)$ is the surface conductivity of the CNT. And then,

$$\sigma_{NW}(z,\omega) = \sigma_{CNT}(z,\omega)\frac{\mathrm{P}_{CNT}}{\Phi_{CNT}} \tag{5}$$

where $\mathrm{P}_{CNT}$ is the perimeter of CNT and $\Phi_{CNT}$ is the area of the cross-section of the CNT.

Considering a NW array, the volume fraction of NWs $\delta_{NW}$, is equal to the proportion of cross-sectional area occupied by the NWs,

$$\delta_{NW} = \frac{N_{NW}\Phi_{NW}}{\Phi_{array}} = \Phi_{NW}D_{NW} \tag{6}$$

where $\Phi_{array}$ is the cross-sectional area of the array, $\Phi_{NW}$ is the cross-sectional area of one NW, $\mathrm{N}_{NW}$ is the number of NW in the array, and $\mathrm{D}_{NW}$ is the number of NW per unit area. Therefore, the effective axial conductivity $\sigma_{axial}$ is the average of the conductivities of the NWs and the air ($\sigma_{air} = 0$),

$$\sigma_{array} = \sigma_{NW}\delta_{NW} = \sigma_{CNT}P_{CNT}D_{CNT} \tag{7}$$

where P is the perimeter of the CNTs and D is the number of CNTs per unit area. By introducing the parameters of the CNT arrays used in our experiment, we can obtain the final conductivity.

For the radial conductivity of CNT arrays, it is difficult to determine the precise value. Considering the radial direction is charge insulating in coupled Luttinger-liquid[5], we simplify the radial conductivity to that of the air, similar treatments have also been reported in previous literatures[3, 6, 7].

**Supplementary Note 3. Calculated dispersion relation of CNT array plasmons, hBN phonon polaritons, and the hybrid mode**

Here, we calculated the dispersion relation of hBN using the transfer matrix method, as shown in Supplementary Figure 2. The in-plane and out-of-plane dielectric functions of hBN used in our calculations can both be described by a single Lorentzian line shape,

$$\varepsilon^{\mu} = \varepsilon_{\infty}^{\mu} + \varepsilon_{\infty}^{\mu} \frac{\left(\omega_{LO}^{\mu}\right)^2 - \left(\omega_{TO}^{\mu}\right)^2}{\left(\omega_{TO}^{\mu}\right)^2 - \omega^2 - i\omega\Gamma^{\mu}}, \mu = z, t \quad (8)$$

where $\omega_{TO}$ and $\omega_{LO}$ represent the transverse optical (TO) and longitudinal optical (LO) phonon frequencies, respectively; $\Gamma$ is the damping constant, and the $\varepsilon_{\infty}$ is high-frequency dielectric constant. The results show perfect consistency with existing literature reports[8, 9].

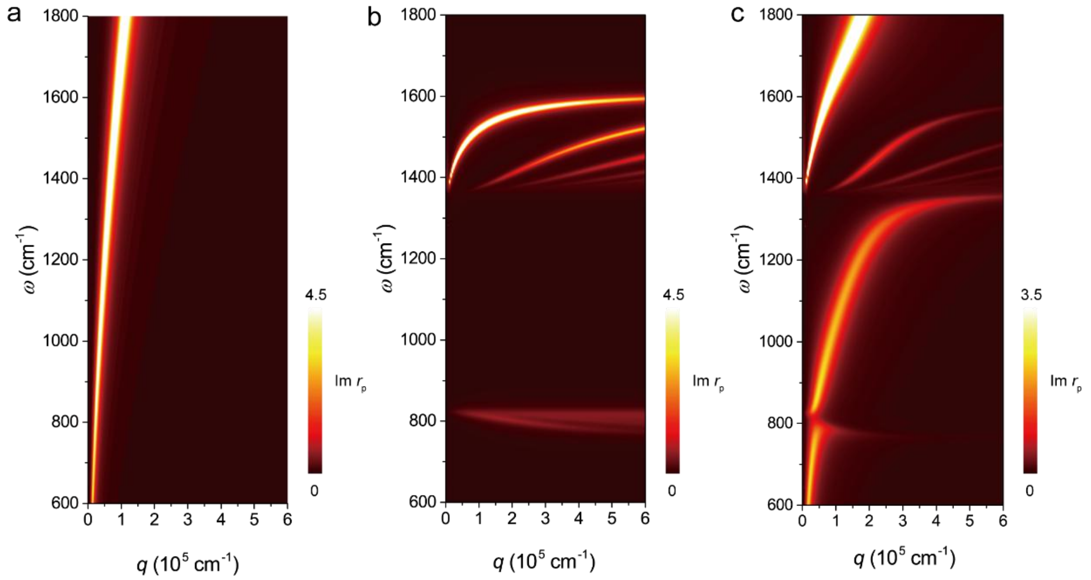


**Supplementary Figure 2. Calculated dispersion relation of the individual carbon nanotube array plasmons, individual hBN phonon polaritons, and their hybrid mode.** a, The dispersion relation of the individual carbon nanotube array plasmons. b, The dispersion relation of the individual hBN phonon polaritons. c, The dispersion relation of the hybrid mode. The calculated dispersion is visualized using a false-color map of the imaginary part of the reflection coefficient $r_p$.

## Supplementary Note 4. Numerical simulations of dispersion relation of hybrid polaritons

Here, we calculated the frequency-momentum dispersion relations of hybrid polaritons using the finite element simulation method (COMSOL), as shown in Supplementary Figure 3. The dispersion relations are similar to Fig.2e, indicating reliable and accurate results.

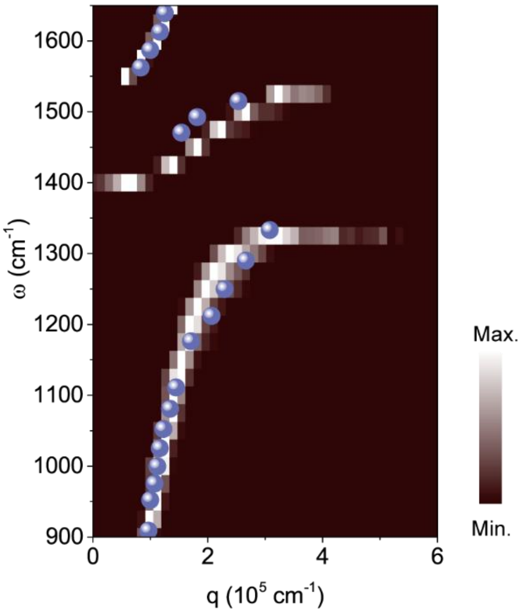


**Supplementary Figure 3. Frequency-momentum dispersion relations of hybrid polaritons in CNT array/hBN heterostructure**. The dots represent the experimental data.

## Supplementary Note 5. IR nano-imaging of topological transition of polaritons reflected by a point defect of a hole

Here, we created a point defect in a CNT array by using AFM etching and then performed IR nano-imaging subsequently, as shown in Supplementary Figure 4. The detailed process of AFM etching can be found in the Methods section. Through precise control of the AFM tip's movement, we reduced the etching path to such a minute scale that the tip appeared stationary. As a result, the etched pattern formed a point-like pit (or a hole).

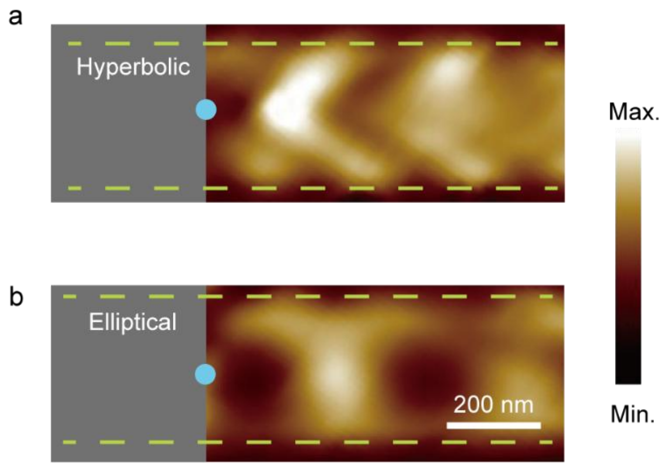


**Supplementary Figure 4. IR nano-imaging of the topological transition of polaritons reflected by a point hole.** **a,** Infrared nano-imaging of hyperbolic polaritons in the CNT array/hBN reflected by a point defect. **b**, Infrared nano-imaging of elliptical polaritons. The blue dot represents the point defect, and the yellow dashed lines represent the boundary of the CNT array. The physical quantity depicted here is the magnitude of the electric field.

## Supplementary Note 6. Numerical simulations of polaritons in CNT array/hBN of different widths

To illustrate the topological transition more clearly, we performed numerical simulations of polaritons on CNT arrays with different widths. Given that our CNT array is size-constrained in the tube radial direction, we demonstrate the polariton patterns in arrays with different numbers of CNTs, as depicted in Supplementary Figure 5. The polaritons were excited at the array boundary (indicated by the dashing line) by an incident plane EM wave polarized along the nanotubes and propagated from left to right. The wavefront of the polaritons tends to converge at a focal point at the frequency of 952 $cm^{-1}$, and the focal length of this point is related to the width of the array—the wider the array, the larger the focal length. At 1575 $cm^{-1}$, the wavefront of the polaritons becomes elliptical and the propagation tends to diverge, resulting in a parallel configuration.

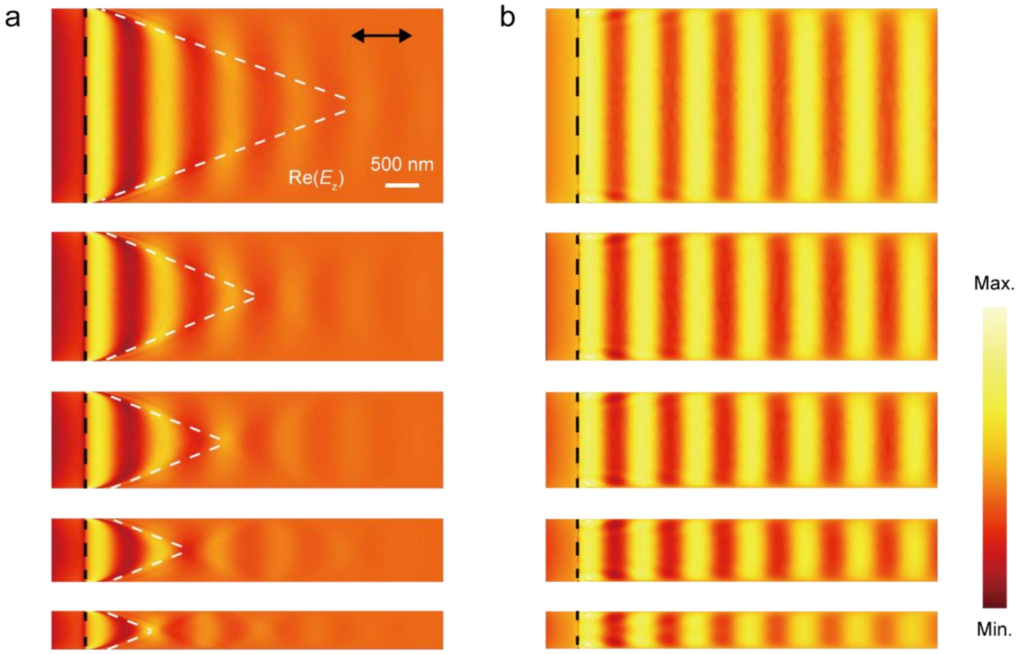


**Supplementary Figure 5. Numerical simulations of polaritons in CNT array/hBN of different widths. a,** Simulated polariton fields in the CNT array/hBN with varying number of CNTs at the frequencies of 952 $cm^{-1}$. **b,** Simulated polariton fields at 1575 $cm^{-1}$. The dashing line represents the terminal boundary of the CNT arrays, while the arrow represents the polarization direction of incident light.

## Supplementary Note 7. The continuous evolution of iso-frequency contours across the transition region.

Here, we calculated the evolution of the iso-frequency contours for hybrid polaritons in CNT-array/hBN across different frequencies as shown in Supplementary Figure 6 below, demonstrating the full process of the polariton topological transition from elliptical to hyperbolic.

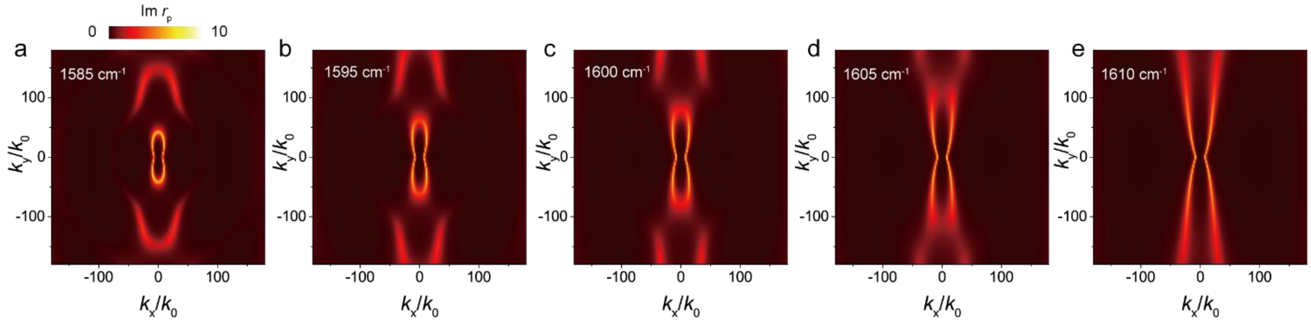


**Supplementary Figure 6. The continuous evolution of iso-frequency contours across the transition region.** The excitation frequencies are 1585$cm^{-1}$ (a), 1595$cm^{-1}$ (b), 1600$cm^{-1}$ (c), 1605 $cm^{-1}$ (d) and 1610$cm^{-1}$ (e), respectively.

## Supplementary Note 8. The evolution of the whispering-gallery mode of the hybrid polaritons with frequency

Here, we provided the frequency-dependent evolution of the hybrid polariton whispering-gallery mode for the sample in Fig. 4 as complementary data, which is depicted in Supplementary Figure 7 below.

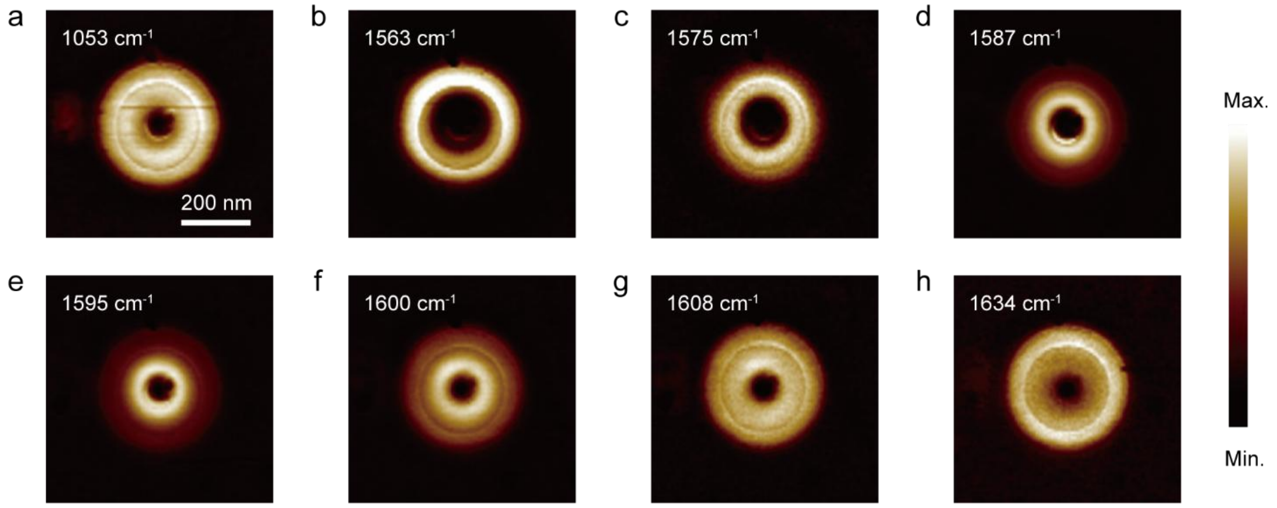


**Supplementary Figure 7. The evolution of the whispering-gallery mode of the hybrid polaritons with frequency in a closed-loop CNT array ring on hBN substrate. a-h**, The infrared nano-imaging of polaritons in the CNT array/hBN at a varying frequency. The extracted quantity of nano-imaging represents the magnitude of the electric field.

## Supplementary Note 9. An additional sample showing the whispering-gallery mode of hybrid polaritons

Here, we demonstrated another CNT ring also exhibits similar plasmonic resonating behavior. The first image in Supplementary Figure 8a is the AFM topography of an additional closed-loop CNT array ring on hBN and the others are the infrared nano-imaging of plasmons in the CNT array/hBN at varying frequency. Near-field profiles of the sections marked by the dashed line is plotted in Supplementary Figure 8b. Notably, the distance between the two constructive plasmon interference peaks exhibits clear oscillations with frequency, demonstrating the resonance of plasmon waves in nanotube rings of varying diameters. Additionally, the intensity of the constructive interference peak also oscillates with

frequency. These features are similar to the sample shown in the Fig. 4 of the manuscript.

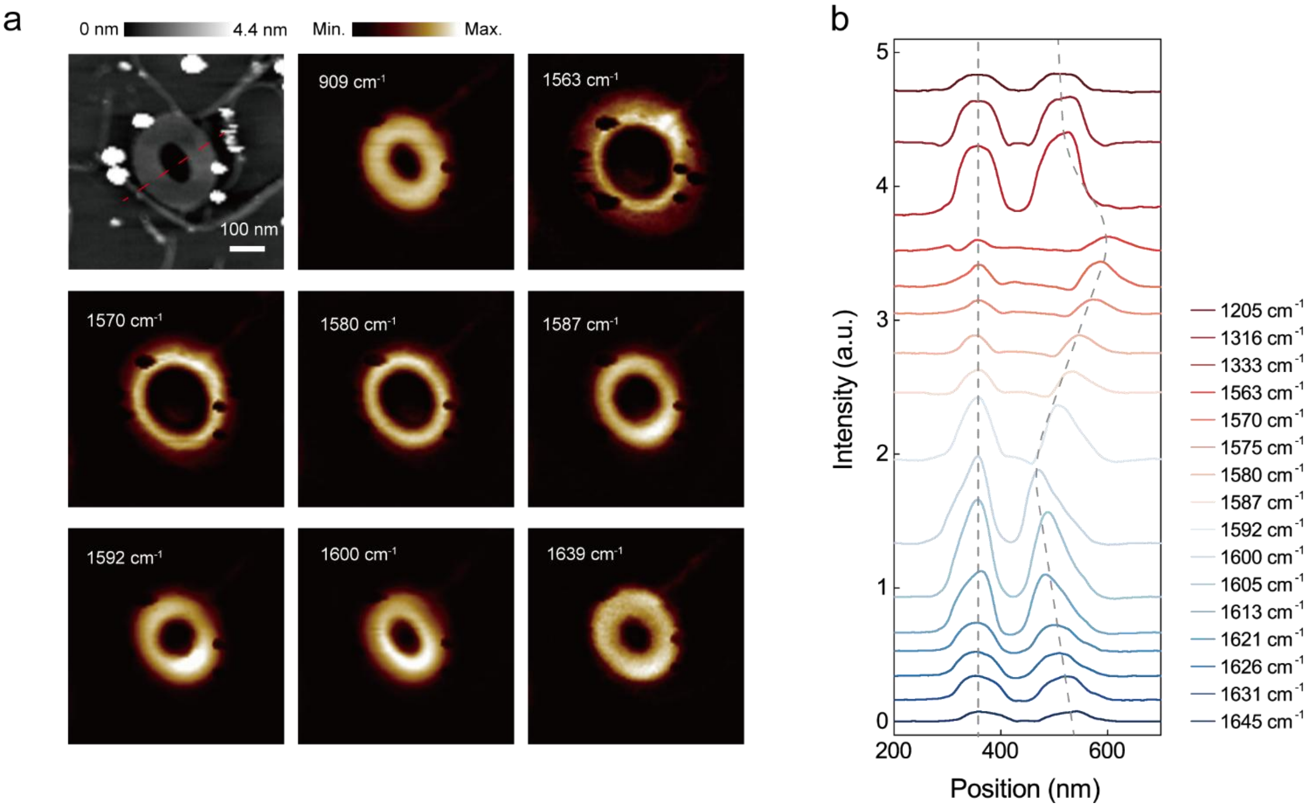


**Supplementary Figure 8. An additional example of whispering-gallery mode in a closed-loop CNT array ring on hBN substrate. a**, AFM topography image of a closed-loop CNT array ring on hBN and the infrared nano-imaging of polaritons in the CNT array/hBN at varying frequency. The extracted quantity of nano-imaging represents the magnitude of the electric field. **b**, Near-field profiles of the sections marked by the dashed line in (a). The physical quantity of the infrared nano-imaging is the near-field intensity.

## Supplementary Note 10. The quality factor of hybrid polaritons in closed-loop CNT array ring on hBN.

Here, we have collected frequency-dependent near-field signal intensity with the background subtracted from a specific point (denoted by a blue dot in Supplementary Fig. 9a) on the closed-loop CNT-array ring on hBN. The results (see Supplementary Fig. 9b), which exhibit a strong fit to a standard resonance peak curve, confirm the presence of a whispering-gallery mode. From this fitting, we extracted the quality factor (Q) as follows: $Q = \frac{\omega_c}{\Delta\omega} = \frac{1600.16\ cm^{-1}}{23.66\ cm^{-1}} = 67.63$, where $\omega_c$ is the resonance frequency and $\Delta\omega$ is the full width at half maximum (FWHM) of the resonance peak. We

now explicitly describe the extracted value as the effective Q-factor under our specific near-field excitation and detection conditions, rather than the intrinsic material Q. We clarify that the linewidth is determined by both the round-trip attenuation and the phase-matching. Therefore, the Q-factor extracted from the FWHM provides an upper-bound estimate of the lifetime of the circulating hybrid polariton mode.

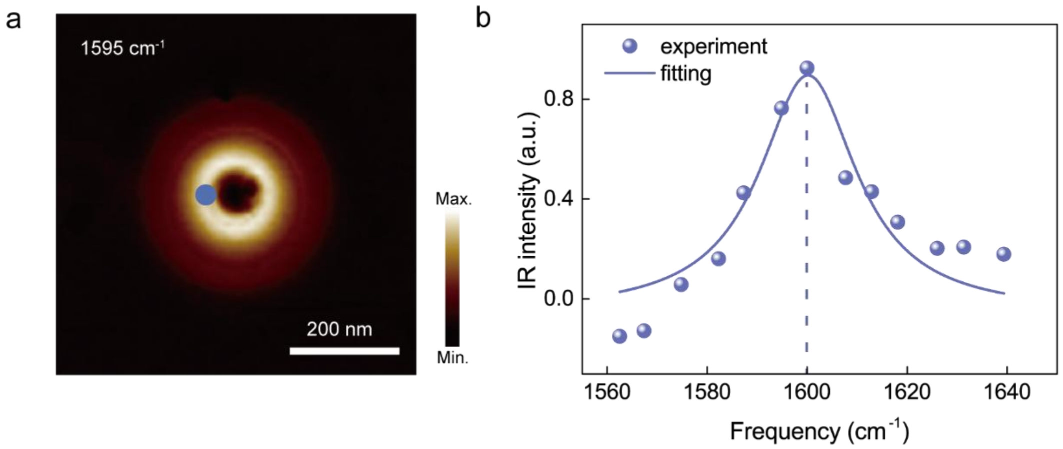


**Supplementary Figure 9. Extraction of the quality factor of the whispery-gallery mode polaritons in closed-loop CNT array/hBN. a,** A typical infrared nano-imaging of polaritons in the CNT-array/hBN. The extracted quantity represents the magnitude of the electric field. **b,** Frequency-dependent near-field signal intensity at a certain point on the closed-loop CNT-array ring on hBN. The position where the experimental data were extracted from is denoted by a blue dot in (a).

## Supplementary Note 11. The evolution of the hybrid polaritons in a larger closed-loop CNT array ring on hBN.

To further support our claim that these are indeed whispering-gallery modes propagating along the axial direction of the CNT array—instead of radial propagation modes— we have selected a larger closed-loop CNT array ring for analysis. The results, presented in Supplementary Fig. 10, illustrate that the path length for polaritons to complete one loop (i.e., the circumference of the circle) is significantly longer, far exceeding the propagation distance of the polaritons. As a result, no constructive or destructive interferences are observed in this configuration.

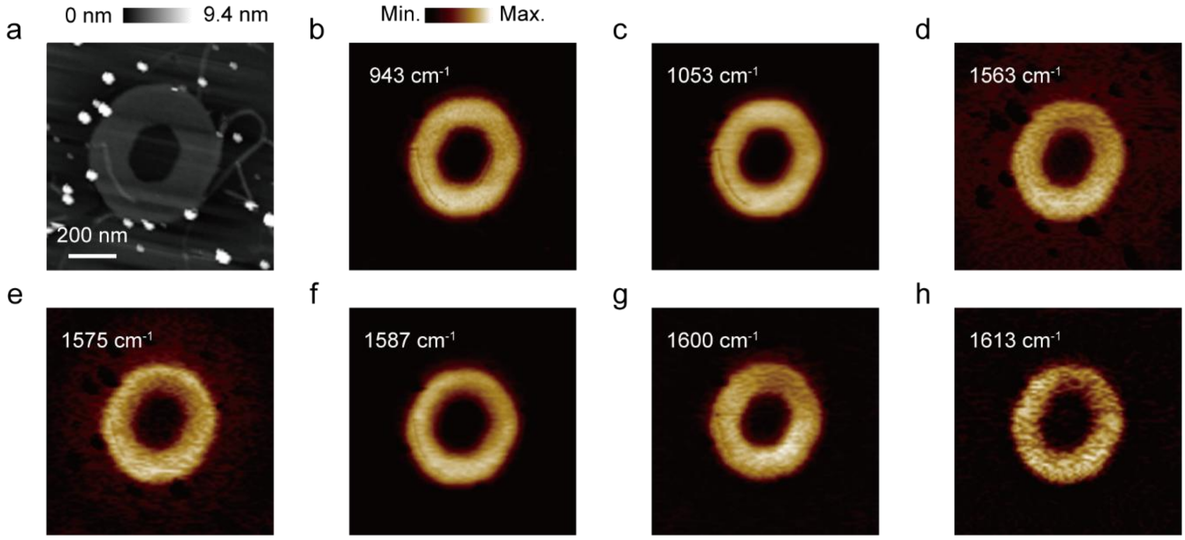


**Supplementary Figure 10. The evolution of the hybrid polaritons in a larger closed-loop CNT array ring on hBN with the excitation frequency, displaying nearly no interference. a,** AFM topography image of a closed-loop CNT array ring on hBN. **b-h,** Infrared nano-imaging of plasmons in the CNT-array/hBN at different frequencies.

## Supplementary References


1. Zhang, Z. et al. Homochiral carbon nanotube van der Waals crystals. *Science* **387**, 1310-1316 (2025).
2. Slepyan, G. Y., Maksimenko, S. A., Lakhtakia, A., Yevtushenko, O. & Gusakov, A. V. Electrodynamics of carbon nanotubes: Dynamic conductivity, impedance boundary conditions, and surface wave propagation. *Physical Review B* **60**, 17136-17149 (1999).
3. Franck, P., Baillargeat, D. & Tay, B. K. Mesoscopic Model for the Electromagnetic Properties of Arrays of Nanotubes and Nanowires: A Bulk Equivalent Approach. *IEEE Transactions on Nanotechnology* **11**, 964-974 (2012).
4. Saiqun, M. et al. Hyperbolic Plasmons in Coupled Luttinger Liquids of Homochiral Carbon Nanotube Arrays. *Chin Phys Lett* **42**, 090710 (2025).
5. Vishwanath, A. & Carpentier, D. Two-Dimensional Anisotropic Non-Fermi-Liquid Phase of Coupled Luttinger Liquids. *Physical Review Letters* **86**, 676-679 (2001).
6. Mikki, S. M. & Kishk, A. A. Mean-Field Electrodynamic Theory of Aligned Carbon Nanotube Composites. *IEEE Transactions on Antennas and Propagation* **57**, 1412-1419 (2009).
7. Nefedov, I. S. Electromagnetic waves propagating in a periodic array of parallel metallic carbon nanotubes. *Physical Review B* **82**, 155423 (2010).
8. Dai, S. et al. Tunable Phonon Polaritons in Atomically Thin van der Waals Crystals of Boron Nitride. *Science* **343**, 1125-1129 (2014).
9. Dai, S. et al. Graphene on hexagonal boron nitride as a tunable hyperbolic metamaterial. *Nature Nanotechnology* **10**, 682-686 (2015).
