# Supplementary material for: Frequency-dependent topological polaritons in carbon nanotube array/hBN heterostructures
Source: Nat Commun. 2026 Mar 24;17:5181. doi: 10.1038/s41467-026-71100-z (PMC13254115; doi:10.1038/s41467-026-71100-z)
Supplement: Supplementary file 2 — Transparent Peer Review file [file 41467_2026_71100_MOESM2_ESM.pdf]

nature portfolio

Peer Review File

Frequency-dependent topological polaritons in carbon nanotube array/hBN heterostructures

Corresponding Author: Professor Zhiwen Shi

Parts of this Peer Review File have been redacted as indicated to remove third-party material.

This file contains all reviewer reports in order by version, followed by all author rebuttals in order by version.

Attachments originally included by the reviewers as part of their assessment can be found at the end of this file.

Version 0:

Reviewer comments:

Reviewer #1

(Remarks to the Author)
In their work, “Configurable plasmon polaritons in carbon nanotube array/hBN heterostructures”, Y. Xie et al. report on coupled polaritons in heterostructures of carbon nanotubes (CNT) arrays and hexagonal boron nitride (hBN). They carefully study a frequency-dependent transition of the polaritons from hyperbolic to elliptical propagation and show indications of a whispering-gallery plasmonic mode in a closed-loop CNT array structure. I feel their work is particularly interesting as it explores polaritons of a relatively new material structure (CNT arrays), which here are hybridized with hBN phonon polaritons.

Overall, I think the results are interesting, novel, and worth publishing. It appears that most of the findings presented are well-supported by an instructive combination of experimental results, numerical simulations, and comparison with literature. While the final section of the manuscript — on whispering-gallery modes — presents interesting findings, it does not yet match the quality and clarity of the rest of the manuscript and should be revised before publication.

Please find below some detailed comments:
1. The manuscript presents experimental results in a relatively broad spectral range from around 900 to >1600 cm-1, which requires a tunable or broadband light source. However, the methods section mentions that the results have been obtained with a CO2 laser at a single wavelength of 10.6 um. Please add relevant information on the laser used for the experiments.

2. As shown in Fig. 1b and described in Supplementary Note 2, the authors simply assume the conductivity of air for the radial direction (y-direction) of the CNT permittivity. While they state that this is consistent with previous research on CNT arrays, the authors do not provide specific references. The permittivity is an essential assumption that should be discussed in more detail. If the permittivity along the y-direction was in fact slightly negative, would this mean that the behavior qualitatively more resembled the well-studied case of graphene and the “topological transition” with frequency may disappear?

3. Regarding the whispering-gallery modes, the authors mention “the intensity of the constructive interference peaks also oscillates with changes in frequency, revealing an inverse relationship between peak spacing and intensity: the smaller the peak spacing, the higher the intensity”. However, Fig. 4d shows “IR intensity” in arbitrary units. How is “intensity” defined here? I.e., how do the authors make sure that their definition of “intensity” is consistent across different frequencies and a frequency-dependent comparison of “intensity” is useful?

4. While all figures in the main manuscript use false-color maps, none of these includes a color bar. It would be helpful to either include color bars for all false-color maps or at least provide a more detailed description of the displayed quantities in the caption.

5. The section on the on the whispering-gallery modes appears to include several claims that are not well supported. Specifically, the following claims should be supported by a quantitative analysis:

a. The authors mention “near-atomic-level smooth, lossless ring-shaped interfaces”. While “lossless” is a strong claim, no quantitative analysis of losses is provided.
b. “The high compression ratio (…) preserves quantum state coherence”. “quantum state coherence” appears vague here and it is not clear how this statement is supported by the data.
c. The authors mention that their structures “showcase the unique benefits of Luttinger liquid plasmons”. This should be discussed in more detail. Specifically, how are the benefits “unique” compared to other plasmonic systems?
d. The authors mention “Compared to traditional low-dimensional plasmonic whispering-gallery nanocavities38, its characteristic dimensions and performance have improved by nearly an order of magnitude.” They refer to reference 38 here. As the authors claim that the performance has increased by nearly an order of magnitude, this should be quantified by comparing suitable figures of merit such as the quality factor. At first glance, the whispering gallery modes shown in Fig. 4 do not appear to have a significantly higher quality compared to the ones shown in reference 38, making a quantitative comparison important.
e. It might be helpful to support the experimental findings on the whispering-gallery modes in Fig. 4 with numerical simulations (e.g., similar to those provided in Fig. 3).

Reviewer #2

(Remarks to the Author)
This manuscript presents an approach to modulate the dispersion of hyperbolic Luttinger-liquid plasmon polaritons (HLLPPs) in carbon nanotube (CNT) arrays through coupling with phonon polaritons (PhPs) in hexagonal boron nitride (h-BN). The authors demonstrate a frequency-controlled topological transition from hyperbolic to elliptic polariton wavefronts and report the observation of a whispering-gallery confined plasmonic mode in the coupled CNT/h-BN heterostructure. The study addresses a topic of clear relevance to the fields of nanophotonics and polaritonics, potentially contributing to the development of tunable hybrid polaritonic systems. However, in my opinion, this manuscript in its current form requires major revisions to become acceptable for publication in Nature Communications.

First, the novelty and physical interpretation need to be clarified, several claims must be quantitatively supported, and the results should be better addressed considering previous works. In addition, several figures and methodological descriptions require improved clarity. Addressing these issues would significantly enhance the scientific quality and overall impact of the manuscript. Please, find detailed comments below.

1. The authors should carefully revise the manuscript to ensure consistent and precise use of polaritonic terminology. Terms such as “plasmon polaritons”, “hybrid polaritons”, and “phonon polaritons” appear to be used interchangeably, which can lead to conceptual ambiguity. Each polariton type should be clearly defined and used consistently according to its physical origin and distinctive characteristics.

2. In Supplementary Information Note 4, the authors state: “The dispersion relations are similar to Fig. 3e, indicating reliable and accurate results.” This appears instead to refer to Figure 2e. The authors should verify the accuracy of all internal references (figures, tables, and text sections) throughout the manuscript.

3. In the Introduction, the authors compare plasmon polaritons in CNTs with phonon polaritons in materials such as h-BN and α-MoO_3. They mention that plasmon polaritons exhibit superior properties, including low loss and broad spectral range. This claim should be quantitatively supported with comparative metrics such as propagation length, lifetime, and confinement, or otherwise tempered. Moreover, phonon polariton systems, though spectrally limited to the Reststrahlen bands, are typically characterized by longer lifetimes and reduced losses compared to plasmonic systems due to their distinct scattering mechanisms. I would recommend the authors to reference prior reports on controlled polariton propagation in phonon-polariton systems (Nano Lett. 2020, 20, 5301–5308; Nano Lett. 2020, 20, 5323–5329; Nat Commun 2024, 15, 9042).

4. The authors report a topological transition of hybrid polaritons from hyperbolic to elliptic wavefronts. Such transitions have been extensively investigated in recent literature, as they can lead to the emergence of the so-called canalized propagation regime. It would be expected that this system also exhibits polariton canalization at the precise topological transition frequency (Optica 2025, 12, 343-349; Nano Lett. 2025, 25, 6, 2203–2209). The authors should discuss whether such canalized regime is expected in the topological transition observed in the CNT array/h-BN system. Also, the qualitative explanation of the hyperbolic-to-elliptic transition based on the sign change of hBN’s in-plane permittivity is reasonable and consistent with established polaritonic behavior. However, the authors show only two discrete frequency cases. A continuous evolution of isofrequency contours across the transition region would more convincingly demonstrate the claimed topological change.

5. The authors describe the observed phenomena as resulting from “strong coupling” between CNT plasmons and h-BN phonon polaritons yet no quantitative coupling parameters (e.g., mode splitting, coupling rate, or linewidth analysis) are provided. This statement should be substantiated with a detailed discussion of the mode splitting visible in the dispersion (Fig. 2e). For completeness, I recommend including the individual dispersions of the CNT array and of h-BN (plots shown in the Supplementary Information), alongside their coupled dispersion, to help the reader clearly visualize the hybridization effect.

6. In Figure 1b, the notation “x4” is unclear. Please define this multiplier explicitly. The authors should also indicate on this panel the excitation frequencies corresponding to the simulated distributions shown in panels c and d.

7. In Figure 1, all panels should include scale bars. If the same scale applies to multiple panels, this should be clearly stated in the caption. Panels c and d should include a shared colorbar indicating the plotted quantity (Re(Ez)), and panels e and f should include a grayscale colorbar for the corresponding magnitude. Please, see, for example, (Nanophotonics 2024, 13, 4761–4770). The use of the term “measured” in the caption is misleading, as Figure 1 presents simulated data. Please replace it with a term such as “calculated” or “simulated”.

8. In Figure 2e, the authors should explain the discrepancy observed near 1400 cm-1 in the experimental data points (high-order mode). Additionally, it would strengthen the analysis to include error bars in the experimental estimations (blue dots) and overlay the analytical dispersion (for example, with a dashed line) on the colorplot derived from the transfer-matrix model for comparison.

9. In the experimental measurement panels (Figure 2c, Figure 3a, Figure 4c, Supplementary Figures 4a,b, and Supplementary Figure 6a), the authors should add a colorbar and clearly specify which experimentally extracted quantity is plotted in each case (amplitude, phase). Was the signal demodulated at higher harmonics? If so, up to which harmonic order?

10. In Figure 3a,b, the authors present an experimental-simulation comparison. First, as previously noted, the authors should indicate which experimentally obtained magnitude is plotted in Figure 3a. Second, please, ensure that the aspect ratios of the experimental and simulated subpanels are identical for a fair visual comparison. Third, I assume that in Figure 3b the authors plot the simulated Re(Ez) … if so, this should be explicitly stated. The authors should note that the only physically meaningful magnitude comparable to Re(Ez) is the real part of the near-field signal, Re(σ_n). If the experiment cannot independently and simultaneously extract s_n and ϕ_n, the only valid comparison with simulations would be between |Ez| and s_n. Finally, this experiment-simulation correspondence could be made clearer by comparing field profiles. Since the dispersion is already shown in Figure 2e, the authors could overlay the experimental data points on the isofrequency contour (IFC) plots in Figure 3c,d as another direct comparison.

11. The authors state: “In this study, we focus exclusively on the analysis of polaritons at frequencies around the type-II band.” To strengthen this discussion, they should include numerical simulations showing the spatial distributions of Re(Ez) across three distinct regions: i) the frequency range where CNT plasmons does not intersect with h-BN phonon polaritons; ii) the frequency range where CNT plasmons intersect with h-BN type I RB phonon polaritons; and iii) the frequency range where CNT plasmons intersect with h-BN type II RB phonon polaritons. What do the authors expect to observe in s-SNOM nano-imaging for type I RB frequencies in this system?

12. Are the authors able to grow or transfer the CNT array onto different substrates (e.g. BaF2 or SiO2) and perform nano-imaging measurements there? I believe it would be highly beneficial for the manuscript to show a direct experimental and numerical comparison between the coupled system (CNT arrays/h-BN) and its individual constituents (CNT array on one side, h-BN on the other) including dispersion plots, Re(Ez) simulations, and s-SNOM measurements.

13. Regarding the sample fabrication process, the authors could clarify whether CNT arrays could be integrated with other 2D van der Waals materials such as graphene, α-MoO3 or α-V2O5.

14. The coupling strength and dispersion modification in plasmon–phonon systems are known to depend sensitively on the hBN thickness. The authors mention using a 55 nm-thick flake “to reduce phonon intensity,” but no data or criteria are shown to justify this choice or demonstrate systematic thickness dependence. I believe that presenting simulations or experiments across several hBN thicknesses would greatly strengthen the coupling interpretation.

15. As pointed out in previous comments, the comparison between experiment and simulation throughout the work is mostly qualitative (“good agreement”). The authors should provide quantitative comparisons to substantiate the agreement. Also, regarding the experimental results, several statements refer to “low loss” “long lifetime”, and “high-quality factor” without quantitative support. Extracted propagation lengths or damping constants from s-SNOM data would be welcome and allow comparison with other polaritonic materials.

16. The interpretation of the observed near-field patterns in Figure 4 as whispering-gallery modes remains highly speculative. The near-field images presented in Figure 4 and the Supplementary Information are consistent with frequency-dependent interference effects, but they do not constitute conclusive evidence of resonant mode confinement. To substantiate the claim of whispering-gallery plasmonic modes, the authors should (i) perform full-wave electromagnetic simulations of the circular CNT-array/hBN structure to visualize the modal field distributions and confirm the existence of azimuthally confined resonances; (ii) provide frequency-dependent spectral data from s-SNOM amplitude or phase measurements to identify discrete resonance peaks; and (iii) estimate the corresponding quality factors (Q) to demonstrate true cavity behavior. Additionally, a systematic comparison across rings of different diameters would enable verification of the expected quantized angular momentum dependence (mode number scaling with circumference). Without such quantitative analysis and modeling, the evidence remains insufficient to support the claim of an “on-chip whispering-gallery nanocavity” and the observed oscillations could alternatively arise from simple standing-wave interference in the hybrid polariton field.

17. The Discussion section includes numerous overstatements (“approached physical limits”, “unprecedented capabilities”, “lays a solid foundation”). These claims should be revised or supported by quantitative metrics.

Reviewer #3

(Remarks to the Author)
See attachment.

[Editorial Note: See end of file]

Reviewer #4

(Remarks to the Author)
I co-reviewed this manuscript with one of the reviewers who provided the listed reports. This is part of the Nature Communications initiative to facilitate training in peer review and to provide appropriate recognition for Early Career Researchers who co-review manuscripts.

Version 1:

Reviewer comments:

Reviewer #1

(Remarks to the Author)
The authors addressed my comments and could resolve most issues. While the revision has improved and strengthened the manuscript and the manuscript contains novel and interesting results worth publishing, some inaccuracies remain. Particularly, the comments related to the whispering-gallery modes (previous comment 5) have not fully been resolved.

Please find some detailed comments below:
1. The abstract now mentions “configurable plasmon frequency-dependent topological polaritons”. I assume this does not indicate any topological protection but simply the transition between the open and closed-loop shape of the IFC, is this correct? If so, this could be clarified.
2. The authors now mention “as the two interference peaks move closer together, the interference pattern becomes more strongly constructive”. The authors could further clarify how “constructiveness” can be quantified/seen from Fig. 4c.
3. Regarding the whispering-gallery modes, the added comparison with the numerical simulations is helpful. However, some comments are still not sufficiently clear. In their response and revised supplementary information, the authors mention “The results (see Supplementary Fig. 9b), which exhibit a strong fit to a standard resonance peak curve, confirm the presence of a whispering-gallery mode.” It is not clear to me why “a strong fit to a standard resonance peak curve” should indicate whispering-gallery mode polaritons. Rather a “standard” resonance peak curve should have various possible explanations, with whispering-gallery mode polaritons being only one of many. As such, the authors should clarify which parts of the results confirm the presence of a whispering-gallery mode.
4. The claims on the whispering-gallery modes should generally be weakened, e.g., using weaker wording, or additional discussion should be included in the manuscript and/or supplementary information. For example, parts of the response to the different reviewers’ comments might be included in the manuscript/SI instead of in the rebuttal letter only, e.g., Fig. R16 and corresponding discussion.
5. Finally, the authors still state that “Compared to traditional low-dimensional polaritonic whispering-gallery nanocavities, its characteristic dimensions and performance have improved by nearly an order of magnitude.” In their rebuttal, the authors indicate that the claimed improvements mainly refer to the critical dimensions. However, the wording in the authors’ manuscript still indicates a broader “performance” improvement by “nearly an order of magnitude”. The authors should either clarify, in the manuscript, that the improvement solely refers to the mode volume (or even just to the physical dimension), or they should detail which other performance improvements they refer to compared to previous work.

Reviewer #2

(Remarks to the Author)
The authors have adequately addressed my comments. I recommend publication in its present form.

Reviewer #3

(Remarks to the Author)
The authors have made a significant improvement of the manuscript and clearly addressed most of my previous comments. Before the acceptance of the manuscript, I just a have a few minor points that need to be clarified and would further strengthen the manuscript.

1) Q-factor of 60 derived from the frequency dependent measurements of a cavity is exceptionally high. Can the authors explain why such a high Q-factor is possible? This three-fold enhancement compared to Q-factors of 20 in straight devices derived by the authors is counterintuitive. Either the specific method of extracting Q-factor of 60 is inaccurate or there is an unknown procedure that enhances the Q-factor in a closed-loop CNT array versus a straight CNT array. This point should be clarified.

2) Whispering gallery mode, WGM, scenario is still not fully clear based on the data itself. Could the authors potentially show a more direct visualization of the WGM, e.g., by placing a gold launcher at one location of the (larger) closed-loop array to

visualize the propagating/circulating WGM? Another simple point to check is whether the WGM wavelength between 1563-1593 cm-1 in a resonance condition. Can the authors compare the round-trip distance around the cavity with respect to the mode wavelength, and confirm if the resonance condition matches the experimental observations?

Reviewer #4

(Remarks to the Author)
I co-reviewed this manuscript with one of the reviewers who provided the listed reports. This is part of the Nature Communications initiative to facilitate training in peer review and to provide appropriate recognition for Early Career Researchers who co-review manuscripts.

Version 2:

Reviewer comments:

Reviewer #1

(Remarks to the Author)
The authors addressed all my comments, and the manuscript appears publishable without further revision.

Reviewer #3

(Remarks to the Author)
While the reviewer agrees that hybridization with hBN phonon polaritons can enhance Q-factor, the authors need to show quantitative evidence of Q-factor enhancement up to 60. Since high quality factor WGM is one of the central claims of the paper, emphasized both in abstract and main text, these points need to be clarified and the derivation and analysis of Q-factor needs to be more rigorously presented.

Besides, based on the additional simulations provided by the authors (Figure R2), the peak in their spectral evolution of near-field signal in a fixed on position within WGM cavity appears to be related to a geometric effect. Near field amplitude under the tip results from tip-launched polaritons under the tip interfering with those making a round-trip across the circumference of cavity. Changing frequency changes WGM wavelength, which shifts the phase of round-trip polaritons with respect to those launched under the tip. When frequency is continuously swept, this continuous increase in the relative phase shift will lead to destructive and to constructive and back to destructive interference, which looks like a peak in spectra. Q-factor derived from the FWHM of the standard fitting to this peak then does not (accurately) reflect the loss of WGM. Can the authors clarify this point?

Reviewer #4

(Remarks to the Author)
I co-reviewed this manuscript with one of the reviewers who provided the listed reports. This is part of the Nature Communications initiative to facilitate training in peer review and to provide appropriate recognition for Early Career Researchers who co-review manuscripts.

Version 3:

Reviewer comments:

Reviewer #3

(Remarks to the Author)
The authors have replied to all the questions I raised. Manuscript can be accepted in the revised form.

Reviewer #4

(Remarks to the Author)
I co-reviewed this manuscript with one of the reviewers who provided the listed reports. This is part of the Nature Communications initiative to facilitate training in peer review and to provide appropriate recognition for Early Career Researchers who co-review manuscripts.

Open Access This Peer Review File is licensed under a Creative Commons Attribution 4.0 International License, which permits use, sharing, adaptation, distribution and reproduction in any medium or format, as long as you give appropriate credit to the original author(s) and the source, provide a link to the Creative Commons license, and indicate if changes were made.
In cases where reviewers are anonymous, credit should be given to 'Anonymous Referee' and the source.
The images or other third party material in this Peer Review File are included in the article's Creative Commons license, unless indicated otherwise in a credit line to the material. If material is not included in the article's Creative Commons license and your intended use is not permitted by statutory regulation or exceeds the permitted use, you will need to obtain permission directly from the copyright holder.
To view a copy of this license, visit https://creativecommons.org/licenses/by/4.0/

Response to Reviewers' Comments (NCOMMS-25-86882)

We sincerely thank all the referees for their careful review of our work and for providing insightful and constructive comments. Below, please find our detailed point-by-point responses.

Response to Reviewer #1

In their work, "Configurable plasmon polaritons in carbon nanotube array/hBN heterostructures", Y. Xie et al. report on coupled polaritons in heterostructures of carbon nanotubes (CNT) arrays and hexagonal boron nitride (hBN). They carefully study a frequency-dependent transition of the polaritons from hyperbolic to elliptical propagation and show indications of a whispering-gallery plasmonic mode in a closed-loop CNT array structure. I feel their work is particularly interesting as it explores polaritons of a relatively new material structure (CNT arrays), which here are hybridized with hBN phonon polaritons.

Overall, I think the results are interesting, novel, and worth publishing. It appears that most of the findings presented are well-supported by an instructive combination of experimental results, numerical simulations, and comparison with literature. While the final section of the manuscript — on whispering-gallery modes — presents interesting findings, it does not yet match the quality and clarity of the rest of the manuscript and should be revised before publication.
Please find below some detailed comments.

Our reply: We thank the reviewer for his/her appreciation of our work and for the positive evaluation. We are also very grateful for the constructive comments provided by the reviewer that have helped us improve the manuscript. Below, we address the questions and comments raised by the reviewer one by one.

1. The manuscript presents experimental results in a relatively broad spectral range from around 900 to >1600 cm^{-1}, which requires a tunable or broadband light source. However, the methods section mentions that the results have been obtained with a CO_2 laser at a single wavelength of 10.6 um. Please add relevant information on the laser used for the experiments.

Our reply: We thank the reviewer for pointing out our mistake in the Methods section.

In addition to the CO_2 laser, we also used a quantum cascade laser (QCL) with a tunable frequency ranging from 900 cm^{-1} to 1600 cm^{-1} in our near-field optical measurement. We have revised the *Methods* section of the manuscript and added the relevant information as follows.

"For near-field infrared nano-imaging, we employed a custom-built scattering-type scanning near-

field optical microscope (s-SNOM) that integrates a tapping-mode atomic force microscope (AFM, Bruker Innova) with a CO_2 laser and a Daylight Solution quantum cascade laser (QCL). The system operates by focusing a mid-infrared laser (900~1650cm^{-1}) beam onto the apex of a conductive AFM probe, generating a highly localized enhanced optical field. This near-field interacts with the sample, and the resulting scattered light, which encodes local optical information, is collected in the far field using a mercury cadmium telluride (MCT) detector (KLD-0.1-J1, Kolmar). To suppress background scattering, the collected signal is demodulated using a lock-in amplifier (Zurich Instruments HF2LI). This configuration allows for the simultaneous acquisition of high-resolution near-field optical images alongside topography information during measurements."

2. As shown in Fig. 1b and described in Supplementary Note 2, the authors simply assume the conductivity of air for the radial direction (y-direction) of the CNT permittivity. While they state that this is consistent with previous research on CNT arrays, the authors do not provide specific references. The permittivity is an essential assumption that should be discussed in more detail. If the permittivity along the y-direction was in fact slightly negative, would this mean that the behavior qualitatively more resembled the well-studied case of graphene and the "topological transition" with frequency may disappear?

Our reply: We sincerely thank the reviewer for his/her insightful comments and suggestions.

For the selection of the radial dielectric constant of carbon nanotube (CNT) arrays, we adopted a strategy that combines referencing parameters from the literature with fitting experimental observations. Theoretically, based on the coupled Luttinger liquid theory, CNT arrays exhibit charge-insulating properties in the radial direction [*Phys. Rev. Lett. 86, 676 (2001)*], which aligns with our previous studies on the material properties of CNT arrays [*Science, 387, 1310 (2025)*]. Based on this, we treated the radial direction as a quasi-air medium (insulating assumption). This simplified approach has been widely adopted in the field of CNT array research [*IEEE Trans. Antennas Propag. 57 1412(2009)*] [*Phys. Rev. B 82 155423(2010)*] [*IEEE Trans. Nanotechnol. 11 964(2012)*]. It has been confirmed that this parameter setting achieves excellent agreement in reproducing the CNT hyperbolic experimental phenomena [*Chinese Phys. Lett. 42, 090710 (2025)*] as well as in fitting the topological hybridization experiments in this study, thereby verifying the robustness of the theoretical model.

Regarding the comment on the possibility that the dielectric constant in the y-direction might be slightly negative, we conducted an in-depth analysis. In the mid-infrared range, the response of CNT arrays in the radial (y) direction to external fields is extremely weak (the magnitude of the dielectric constant is very small), and the dominant characteristic of the system is hyperbolic plasmonic behavior. Simulations and corresponding iso-frequency contours (see Fig. R1 below) show that setting the radial dielectric constant to small negative values (e.g., -2) yields results that are nearly identical to those obtained with a value of +1. Physically, while a negative dielectric constant supports high-momentum radial modes (manifested as short-period oscillations), these modes are distinct from the characteristics of axial hyperbolic modes and do not significantly affect the propagation behavior of the hyperbolic wavefront.

It is important to emphasize that only when the absolute value of the radial dielectric constant becomes sufficiently large does the momentum of the radial mode significantly decrease, leading to a topological phase transition from hyperbolic polaritons to elliptical polaritons (as shown in Fig. R1f). Only under such extreme conditions would the frequency-dependent topological transition disappear, and the system would exhibit isotropic behavior similar to graphene. Therefore, within the current parameter range, our conclusions remain robust.

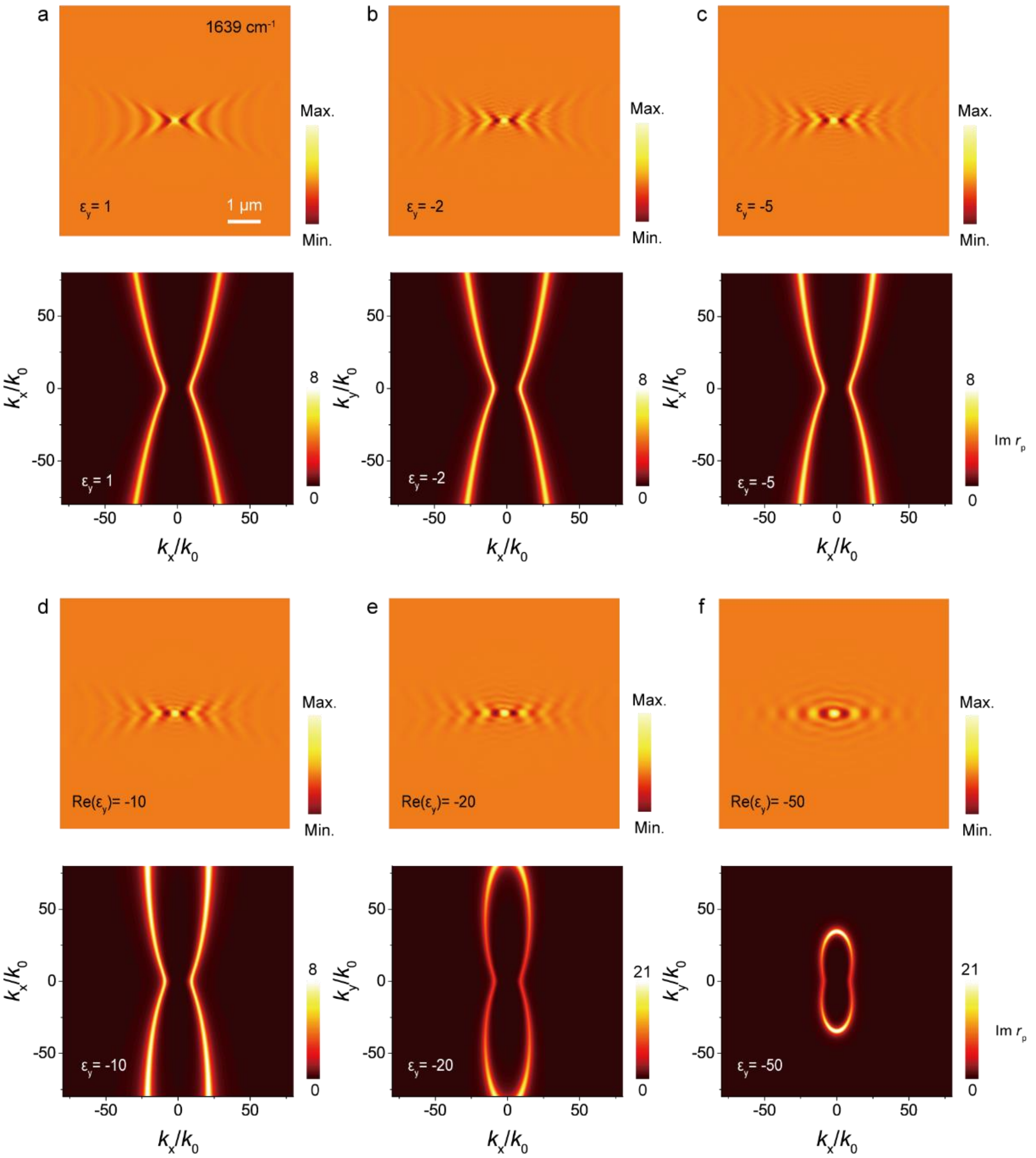

Figure R1. The influence of radial dielectric constant on the topological transition. The numerical simulated spatial distribution of the real part of the polariton field Re(E_z) (top)measured above the CNT-array/hBN at frequencies of 1639cm^{-1} and corresponding iso-frequency contours (bottom) with different radial dielectric constants.

3. Regarding the whispering-gallery modes, the authors mention "the intensity of the constructive interference peaks also oscillates with changes in frequency, revealing an inverse relationship

between peak spacing and intensity: the smaller the peak spacing, the higher the intensity". However, Fig. 4d shows "IR intensity" in arbitrary units. How is "intensity" defined here? I.e., how do the authors make sure that their definition of "intensity" is consistent across different frequencies and a frequency-dependent comparison of "intensity" is useful?

Our reply: We appreciate the reviewer's insightful comments.

In our study, "intensity" is defined as the relative difference between the near-field signal—specifically, the magnitude of the electric field perpendicular to the surface ($|E_z|$)—at the interference-enhanced peak of CNT-array/hBN polaritons and the background level observed in the pure hBN region. Given that hBN forms a substantial part of the substrate, providing an overarching background, we believe this definition of intensity is appropriate. Throughout the experiments, we maintained a consistent intensity of incident light across varying frequencies to ensure comparability.

To enhance clarity, we have refined our statement to:

"As the two interference peaks move closer together, the interference pattern becomes more strongly constructive."

4. While all figures in the main manuscript use false-color maps, none of these includes a color bar. It would be helpful to either include color bars for all false-color maps or at least provide a more detailed description of the displayed quantities in the caption.

Our reply: We thank the reviewer for his/her kind reminder.

In our revised manuscript, we have added color bars to all false-color maps to enhance clarity. Additionally, we have provided more detailed descriptions of the displayed quantities in the figure captions.

5. The section on the on the whispering-gallery modes appears to include several claims that are not well supported. Specifically, the following claims should be supported by a quantitative analysis:
a. The authors mention "near-atomic-level smooth, lossless ring-shaped interfaces". While "lossless" is a strong claim, no quantitative analysis of losses is provided.
b. "The high compression ratio (...) preserves quantum state coherence". "quantum state coherence" appears vague here and it is not clear how this statement is supported by the data.
c. The authors mention that their structures "showcase the unique benefits of Luttinger liquid plasmons". This should be discussed in more detail. Specifically, how are the benefits "unique" compared to other plasmonic systems?
d. The authors mention "Compared to traditional low-dimensional plasmonic whispering-gallery nanocavities38, its characteristic dimensions and performance have improved by nearly an order of magnitude." They refer to reference 38 here. As the authors claim that the performance has

increased by nearly an order of magnitude, this should be quantified by comparing suitable figures of merit such as the quality factor. At first glance, the whispering gallery modes shown in Fig. 4 do not appear to have a significantly higher quality compared to the ones shown in reference 38, making a quantitative comparison important.
e. It might be helpful to support the experimental findings on the whispering-gallery modes in Fig. 4 with numerical simulations (e.g., similar to those provided in Fig. 3).

Our reply: We thank the reviewer for his/her constructive comments. We have addressed each comment and suggestion point by point as outlined below.

a-c: The plasmons in CNTs have been reported to exhibit characteristics of Luttinger liquid plasmons, demonstrating excellent properties of high spatial confinement and high quality factors, as detailed in previous literature [*Nature Photon. 9, 515-519 (2015)*]. We have quantitatively analyzed these properties using the quality factor $Q = Re(q)/Im(q)$ and the compression factor $N = Re(q)/k_0$. Additionally, we fitted the experimental near-field profile with the following formula,

$$I = \left|\eta_s + \eta_R \frac{1}{\sqrt{2x}} e^{\frac{-2x}{a}} e^{i(2qx+\phi_R)}\right|$$

where η_s represents the scattering rate of incident light back into the detector, η_R is the reflectivity of HPs at the edge, q is the plasmon wavevector, *a* is the decay length, and ϕ_R is the reflection phase. This formula is derived from the literature [*Adv. Funct. Mater. 2019, 29, 1904662*].

We selected experimental data at frequencies of 1111 cm^{-1} and 1639 cm^{-1} for fitting, as shown in Fig. R2 below. Notably, the quality factor of the CNT array plasmons reaches up to 21, while the compression factor is 21.57. These results highlight that even structured into whispering-gallery-mode oscillators, CNT plasmons retain their excellent intrinsic properties, significantly lowering optical scattering losses, and maintaining low intrinsic dissipation, thus preserving optical state coherence [*Nano Lett. 2021, 21, 8707–8714*] [*Nature, 2020, 582(7810): 46-49*].

To support this conclusion, we collected frequency-dependent near-field signal intensity data with the background subtracted, from a specific point (denoted by a blue dot in Fig. R3a) on the closed-loop CNT-array ring on hBN. The results (see Fig. R3b), which exhibit a strong fit to a standard resonance peak curve, confirm the presence of a whispering-gallery mode. From this fitting, we extracted the quality factor (Q) as follows: $Q = \frac{\omega_c}{\Delta\omega} = \frac{1600.16\ cm^{-1}}{23.66\ cm^{-1}} = 67.63$, where ω_c is the resonance frequency and $\Delta\omega$ is the full width at half maximum (FWHM) of the resonance peak.

However, we acknowledge that some expressions in our manuscript are not rigorous enough and require further refinement. For example, we have revised "lossless ring-shaped interfaces" to "low-loss ring-shaped interfaces", and "The high compression ratio not only preserves quantum state coherence" to "The high compression ratio and quality factor not only preserve optical state coherence".

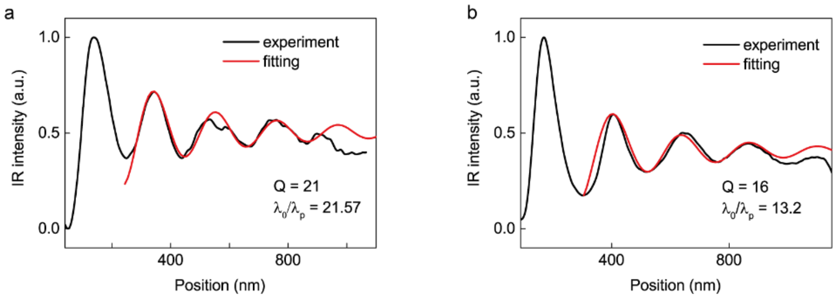

Figure R2. Extraction of the quality factor of plasmons in CNT array. a, The near-field profile along the axial direction of CNT array at the frequency of 1111 cm^{-1} and the corresponding fitting curve. **b,** The near-field profile along the axial direction of CNT array at the frequency of 1639 cm^{-1} and the corresponding fitting curve.

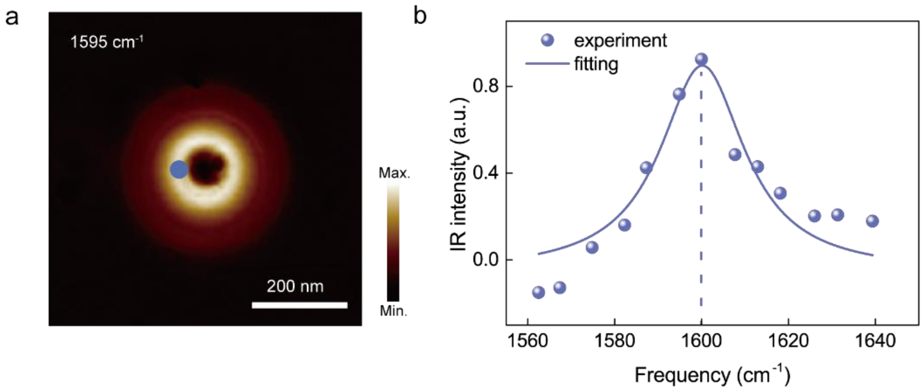

Figure R3. Extraction of the quality factor of the whispery-gallery mode polaritons in closed-loop CNT array/hBN. a, A typical infrared nano-imaging of polaritons in the CNT-array/hBN. **b,** Frequency-dependent near-field signal intensity at a certain point on the closed-loop CNT-array ring on hBN. The position where the experimental data were extracted from is denoted by a blue dot in (a).

d. The term "characteristic dimensions and performance" in the manuscript refers to the mode volume ratio V_p/V_0 , which is approximately one order of magnitude lower than that of the nanocavity reported in reference 38 [*Nat. Mater. 22, 838–843 (2023)*]. This estimation method follows the approach used in previous literatures [*Science 360, 291-295 (2018)*] [*Science 368, 1219-1223 (2020)*], where the polariton wavelength $\lambda_p = \lambda_0/16.5$ is used. Given our CNT nanocavity's thickness is on the atomic scale, roughly 4 nm, we obtain $V_p/V_0 = 2.3 \times 10^{-6}$, which is less than the value of 10^{-5} reported in reference 38 [*Nat. Mater. 22, 838–843 (2023)*].

e. We have conducted numerical simulations of the polariton whispering-gallery mode and incorporated these results into Fig.4 of the revised manuscript (see Fig. R4). Our simulation method involved placing an electric dipole above the resonant ring to mimic the AFM tip to excite the polaritons, and collecting the E_z component below the dipole. The mapping of dipole scanning positions yielded results that correlate well with our experimental findings.

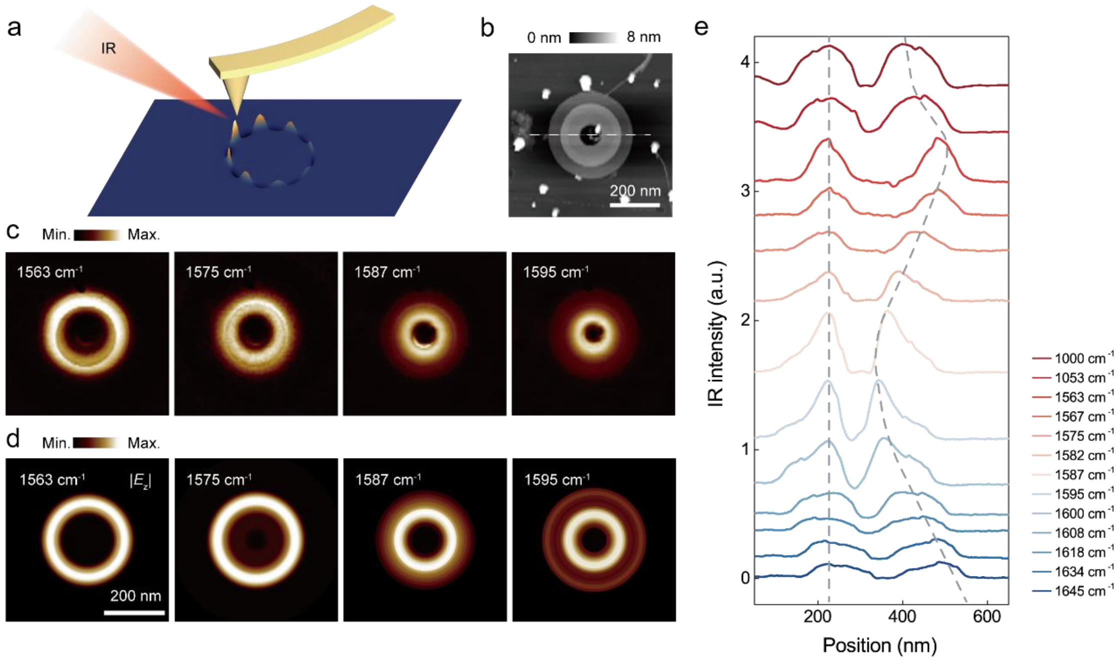

Figure R4. The revised version of Figure 4 of the manuscript. a, Schematic of infrared nano-imaging of whispering-gallery polaritonic mode in the CNT-array/hBN by using s-SNOM. **b,** AFM topography image of a closed-loop CNT array ring on hBN. Infrared nano-imaging experiment (**c**) and corresponding numerical simulations (**d**) of polaritons in the CNT-array/hBN at different frequencies, respectively. **e,** The Near-field line profiles along the sections marked by the dashed line in (b).

Response to Reviewer #2

This manuscript presents an approach to modulate the dispersion of hyperbolic Luttinger-liquid plasmon polaritons (HLLPPs) in carbon nanotube (CNT) arrays through coupling with phonon polaritons (PhPs) in hexagonal boron nitride (h-BN). The authors demonstrate a frequency-controlled topological transition from hyperbolic to elliptic polariton wavefronts and report the observation of a whispering-gallery confined plasmonic mode in the coupled CNT/h-BN heterostructure. The study addresses a topic of clear relevance to the fields of nanophotonics and polaritonics, potentially contributing to the development of tunable hybrid polaritonic systems. However, in my opinion, this manuscript in its current form requires major revisions to become acceptable for publication in Nature Communications.

First, the novelty and physical interpretation need to be clarified, several claims must be quantitatively supported, and the results should be better addressed considering previous works. In addition, several figures and methodological descriptions require improved clarity. Addressing these issues would significantly enhance the scientific quality and overall impact of the manuscript. Please, find detailed comments below.

Our reply: We appreciate the reviewer's recognition of our work and the valuable suggestions for enhancing the manuscript. Below, we address each question and comment in detail.

1. The authors should carefully revise the manuscript to ensure consistent and precise use of polaritonic terminology. Terms such as "plasmon polaritons", "hybrid polaritons", and "phonon polaritons" appear to be used interchangeably, which can lead to conceptual ambiguity. Each polariton type should be clearly defined and used consistently according to its physical origin and distinctive characteristics.

Our reply: We appreciate the reviewer's insightful suggestions regarding the use of polaritonic terminology.

In response, we have revised some terms throughout the manuscript. In particular, we have revised the manuscript title from "*Configurable plasmon polaritons in carbon nanotube array/hBN heterostructures*" to "*Frequency-dependent topological polaritons in carbon nanotube array/hBN heterostructures*" for greater clarity.

To clarify terminology within the manuscript: "plasmon polaritons" refers to the polaritons generated from the interaction between charges in CNT arrays and incident photons, while "phonon polaritons" denote the interactions between phonons in hBN and photons. The term "hybrid polaritons" describes the coupled modes resulting from plasmon and phonon polariton interactions. Specifically, in regions outside the Reststrahlen band 2 of hBN, only plasmon polaritons propagate in the CNT-array/hBN system. However, within the Reststrahlen band, CNT arrays plasmon polaritons are classified as hybrid polaritons due to their coupling with phonon

polaritons in hBN.

To eliminate ambiguity, we have made these definitions consistent throughout the revised manuscript.

2. In Supplementary Information Note 4, the authors state: "The dispersion relations are similar to Fig. 3e, indicating reliable and accurate results." This appears instead to refer to Figure 2e. The authors should verify the accuracy of all internal references (figures, tables, and text sections) throughout the manuscript.

Our reply: We thank the reviewer for his/her careful review, and for pointing out our mistake.

The typo has been corrected, as shown below.

"Here, we calculated the frequency-momentum dispersion relations of hybrid plasmons using the finite element simulation method (COMSOL), as shown in Supplementary Figure 3. The dispersion relations are similar to Fig.2e, indicating reliable and accurate results."

3. In the Introduction, the authors compare plasmon polaritons in CNTs with phonon polaritons in materials such as h-BN and α-MoO_3. They mention that plasmon polaritons exhibit superior properties, including low loss and broad spectral range. This claim should be quantitatively supported with comparative metrics such as propagation length, lifetime, and confinement, or otherwise tempered. Moreover, phonon polariton systems, though spectrally limited to the Reststrahlen bands, are typically characterized by longer lifetimes and reduced losses compared to plasmonic systems due to their distinct scattering mechanisms. I would recommend the authors to reference prior reports on controlled polariton propagation in phonon-polariton systems (Nano Lett. 2020, 20, 5301–5308; Nano Lett. 2020, 20, 5323–5329; Nat Commun 2024, 15, 9042).

Our reply: We appreciate the reviewer's valuable comments regarding the comparative analysis of plasmon polaritons in CNTs and phonon polaritons in materials like h-BN and α-MoO_3.

In our manuscript, we attribute the low loss of CNT plasmons to the suppression of electron backscattering in their one-dimensional structure, allowing Luttinger-liquid plasmons to achieve high quality factors of up to 20 (*Nat. Photon., 9, 515 [2015]*). This quality factor is generally superior to that of plasmons observed on other low-dimensional surfaces, such as graphene. However, we acknowledge that CNT plasmons do not possess this advantage when compared to phonon polaritons.

To enhance clarity and precision, we have revised the introduction with the following statement:

" Plasmon polaritons, particularly in one-dimensional carbon nanotubes (CNTs), exhibit exceptional properties, including deep subwavelength confinement and a broad spectral range."

Furthermore, we have incorporated the references suggested by the reviewer (*Nano Lett. 2020, 20, 5301–5308; Nano Lett. 2020, 20, 5323–5329; Nat Commun 2024, 15, 9042*) as citations in the revised manuscript.

4. The authors report a topological transition of hybrid polaritons from hyperbolic to elliptic wavefronts. Such transitions have been extensively investigated in recent literature, as they can lead to the emergence of the so-called canalized propagation regime. It would be expected that this system also exhibits polariton canalization at the precise topological transition frequency (Optica 2025, 12, 343-349; Nano Lett. 2025, 25, 6, 2203–2209). The authors should discuss whether such canalized regime is expected in the topological transition observed in the CNT array/h-BN system. Also, the qualitative explanation of the hyperbolic-to-elliptic transition based on the sign change of hBN's in-plane permittivity is reasonable and consistent with established polaritonic behavior. However, the authors show only two discrete frequency cases. A continuous evolution of isofrequency contours across the transition region would more convincingly demonstrate the claimed topological change.

Our reply: We appreciate the reviewer's constructive suggestions regarding the topological transition of hybrid polaritons.

The small width of our as-grown CNT arrays (only several hundred nanometers) makes it experimentally challenging to accurately determine the excitation frequency for polariton canalization. To address this, we have supplemented our experimental findings with analytical calculations, as shown in Fig. R5. The results, illustrated in the figure below, show that the polariton iso-frequency contours transition from elliptic to hyperbolic as the excitation frequency increases. This analysis indicates that a canalization state emerges around the frequency of 1605 cm^{-1}.

Furthermore, we have incorporated the Fig. R5 as part of the revised Supplementary Information and added the references suggested by the reviewer (*Optica 2025, 12, 343-349; Nano Lett. 2025, 25, 6, 2203–2209*) as citations in the revised manuscript.

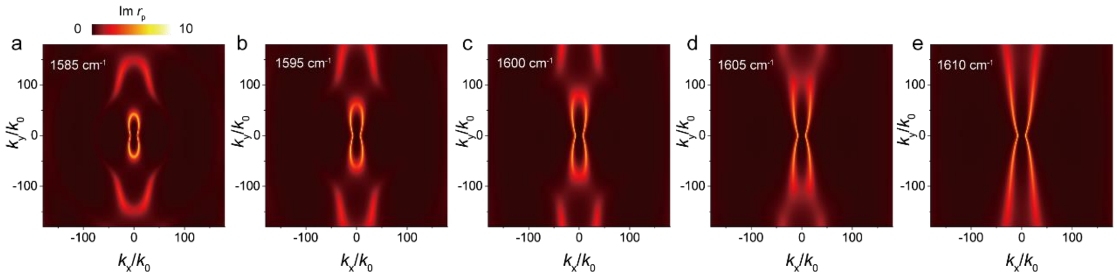

Figure R5. The continuous evolution of iso-frequency contours across the transition region. The excitation frequencies are 1585cm^{-1} (a), 1595cm^{-1} (b), 1600cm^{-1}(c), 1605 cm^{-1} (d) and 1610cm^{-1} (e), respectively.

5. The authors describe the observed phenomena as resulting from "strong coupling" between CNT

plasmons and h-BN phonon polaritons yet no quantitative coupling parameters (e.g., mode splitting, coupling rate, or linewidth analysis) are provided. This statement should be substantiated with a detailed discussion of the mode splitting visible in the dispersion (Fig. 2e). For completeness, I recommend including the individual dispersions of the CNT array and of h-BN (plots shown in the Supplementary Information), alongside their coupled dispersion, to help the reader clearly visualize the hybridization effect.

Our reply: We thank the reviewer for the valuable suggestions regarding the coupling parameters of the observed phenomena.

To quantitatively address the mode splitting of hybrid polaritons in the CNT array/hBN system, we employed a method similar to that used in previous literature [*Nat. Mater. (2025). https://doi.org/10.1038/s41563-025-02412-6*]. The mode splitting of hybrid polaritons in the CNT array/hBN can be expressed as:

$$\omega_{\pm} = \frac{1}{\sqrt{2}}\sqrt{\omega_{spp}^2 + \omega_{php}^2 + 4g^2 \pm \sqrt{(\omega_{spp}^2 + \omega_{php}^2 + 4g^2)^2 - 4\omega_{spp}^2\omega_{php}^2}}$$

where, g is the coupling strength, ω_{spp} and ω_{php} represent the bare modes of CNT array plasmons and hBN phonon polaritons, respectively.

Then, we extracted $\omega_c = 1357\ cm^{-1}$ and $g = \frac{\Delta\omega}{2} = 319.2\ cm^{-1}$ from the calculated dispersion relations, where the ω_c represents the frequency where bare modes cross. Therefore, the normalized coupling strength is calculated as $\frac{g}{(\Gamma_{php}+\Gamma_{spp})/2} = 3.45 > 1$, where $\Gamma_{php} = 10\ cm^{-1}$ and $\Gamma_{spp} = 174.79\ cm^{-1}$ represent the loss rates of hBN phonon polaritons and CNT array plasmons, respectively. This result substantiates that the hybrid mode between CNT array plasmons and hBN phonon polaritons can be classified as strong coupling. [*Nat. Rev. Phys. 1, 19 – 40 (2019)*]

Following the referee's suggestion, we have included a comparative plot of the dispersions for the individual carbon nanotube array plasmons, individual hBN phonon polaritons, and their hybrid mode, as shown in Figure R6 below. This figure is now part of the Supplementary Information.

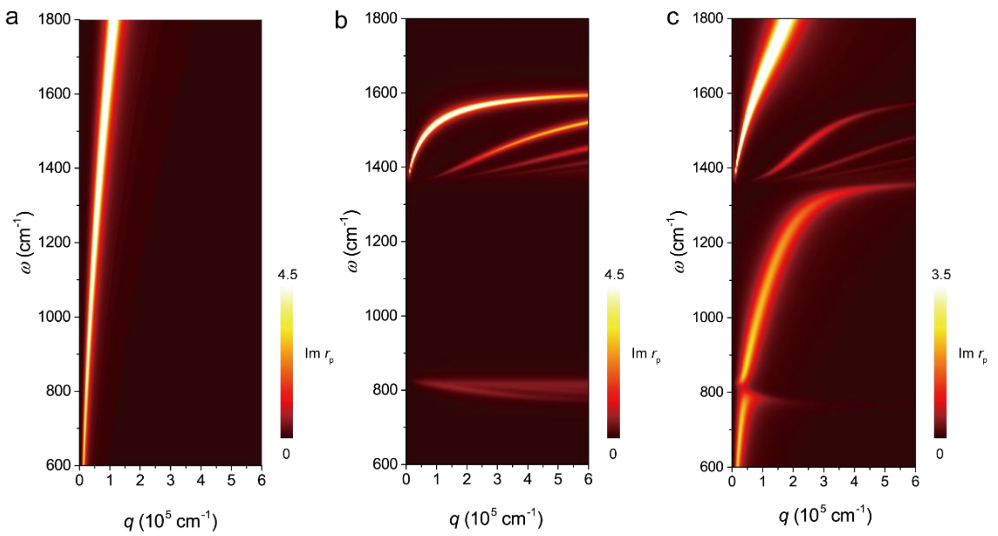

Figure R6. Calculated dispersion relation of the individual carbon nanotube array plasmons, individual hBN phonon polaritons, and their hybrid modes. a, The dispersion relation of the individual carbon nanotube array plasmons. **b,** The dispersion relation of the individual hBN phonon polaritons. **c,** The dispersion relation of the hybrid mode. The calculated dispersion is visualized using a false-color map of the imaginary part of the reflection coefficient r_p.

6. In Figure 1b, the notation "x4" is unclear. Please define this multiplier explicitly. The authors should also indicate on this panel the excitation frequencies corresponding to the simulated distributions shown in panels c and d.

Our reply: We appreciate the reviewer's careful review and valuable feedback.

To enhance clarity, we have added the following note to the caption of Figure 1b:

"*The 'x4' label means that the in-plane permittivity of hBN has been multiplied by four so that the dielectric functions of both hBN and CNTs can be displayed together on the same y-axis.*"

Additionally, we have indicated the excitation frequencies in Figure 1b that correspond to the simulated distributions shown in Figures 1c and 1d of the revised manuscript.

7. In Figure 1, all panels should include scale bars. If the same scale applies to multiple panels, this should be clearly stated in the caption. Panels c and d should include a shared colorbar indicating the plotted quantity (Re(Ez)), and panels e and f should include a grayscale colorbar for the corresponding magnitude. Please, see, for example, (Nanophotonics 2024, 13, 4761–4770). The use of the term "measured" in the caption is misleading, as Figure 1 presents simulated data. Please replace it with a term such as "calculated" or "simulated".

Our reply: We appreciate the reviewer's careful and precise feedback.

In the revised Figure 1, we have included scale bars in all panels. If the same scale applies to multiple panels, this has been clearly stated in the caption. Additionally, we have added a shared color bar for panels c and d, indicating the plotted quantity Re(E_z), and a grayscale color bar for panels e and f to represent the corresponding magnitude.

Furthermore, we have replaced the term “measured” with “simulated” in the caption to accurately reflect the nature of the data presented. Besides, we have incorporated the references suggested by the reviewer (*Nanophotonics 2024, 13, 4761–4770*) as citations in the revised manuscript.

8. In Figure 2e, the authors should explain the discrepancy observed near 1400 cm^{-1} in the experimental data points (high-order mode). Additionally, it would strengthen the analysis to include error bars in the experimental estimations (blue dots) and overlay the analytical dispersion (for example, with a dashed line) on the colorplot derived from the transfer-matrix model for comparison.

Our reply: We appreciate the reviewer’s important suggestions regarding Figure 2e.

The observed discrepancy near 1400 cm^{-1} between the experimental data points and the calculated dispersion relation can be attributed to measurement uncertainties. Specifically, in the frequency region of plasmon-phonon polariton coupling, our experimental signal reflects a superposition of the fundamental and second-order polariton modes. Due to an insufficient number of measurement periods caused by the sample size, the extracted wavelength values may not align perfectly with theoretical calculations.

In the revised manuscript, we have incorporated the following analysis:

“*The discrepancy between the experimental data and the calculated dispersion in the Reststrahlen band arises because our measurement captures a superposition of the fundamental and second-order modes of the hybrid polariton.*”

In response, we measured the polariton wavelength again and quantified the reading uncertainty, with the results plotted in Fig. R7a. As can be seen, the reading error in extracting wave vectors from the experimental data is negligible, rendering error bars virtually invisible on the plot; therefore, they have been omitted. We attribute the observed discrepancy in the Reststrahlen band to the experimental indistinguishability between the fundamental and higher-order modes. Therefore, to ensure optimal visual presentation and avoid readability issues, we have chosen not to include error bars in the experimental dispersion relation. Similar treatment has been employed in many previously reported studies on the coupling of hBN polaritons and graphene plasmons, as depicted in Fig. R8. [*Nature Nanotech. 10, 682–686 (2015)*] [*Nature Mater. 14, 421–425 (2015)*].

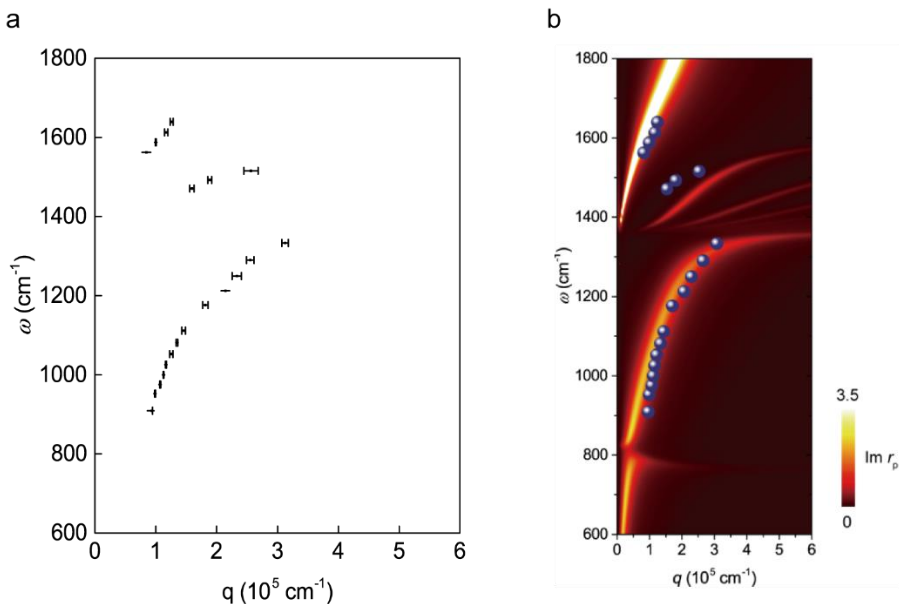

Figure R7. Experimental dispersion relation including error bars. a, Experimental dispersion relation of hybrid polaritons in the CNT-array/hBN, with error bars included. B, Comparison of calculated and experimental dispersion relations taken from the Fig.2e of the manuscript.

[REDACTED]

Figure R8. Experimental dispersion relation of hybrid polaritons in graphene/hBN heterostructure. Corresponding simulation results are also provided as the background in previous literatures. (a) is taken from Fig. 2c of the paper Dai et al. [*Nature Nanotech. 10, 682–686 (2015)*]. (b) is taken from Fig. 2d of the paper Woessner et al. [*Nature Mater. 14, 421–425 (2015)*].

9. In the experimental measurement panels (Figure 2c, Figure 3a, Figure 4c, Supplementary Figures 4a,b, and Supplementary Figure 6a), the authors should add a colorbar and clearly specify which experimentally extracted quantity is plotted in each case (amplitude, phase). Was the signal demodulated at higher harmonics? If so, up to which harmonic order?

Our reply: We appreciate the reviewer's careful review and valuable suggestions.

In the revised manuscript, we have added color bars and provided textual descriptions of the

extracted physical quantities for each experimental measurement panel (Figure 2c, Figure 3a, Figure 4c, Supplementary Figures 4a and 4b, and Supplementary Figure 6a). The extracted quantity represents the magnitude of the electric field, corresponding to the third order near-field signal.

10. In Figure 3a,b, the authors present an experimental-simulation comparison. First, as previously noted, the authors should indicate which experimentally obtained magnitude is plotted in Figure 3a. Second, please, ensure that the aspect ratios of the experimental and simulated subpanels are identical for a fair visual comparison. Third, I assume that in Figure 3b the authors plot the simulated Re(Ez) … if so, this should be explicitly stated. The authors should note that the only physically meaningful magnitude comparable to Re(Ez) is the real part of the near-field signal, Re(σ_n). If the experiment cannot independently and simultaneously extract s_n and ϕ_n, the only valid comparison with simulations would be between |Ez| and s_n. Finally, this experiment-simulation correspondence could be made clearer by comparing field profiles. Since the dispersion is already shown in Figure 2e, the authors could overlay the experimental data points on the isofrequency contour (IFC) plots in Figure 3c,d as another direct comparison.

Our reply: We thank the reviewer for his/her significant comments and insightful suggestions.

The physical quantity depicted in Fig. 3a is the near-field intensity, while Fig. 3b shows $Re(E_z)$. This information has been labeled in the revised manuscript. The experimentally observed pattern arises from the interference between the incident field E_{in} and the polariton field E_p, i.e. $E_z = E_{in} + E_p$, as depicted in Figs. R9 a,b [*eLight 3, 14 (2023)*]. The polariton field forms an interference pattern from its tip-excited and reflected components. Hence, the resulting fringes correspond to the phase information of $Re(E_z)$. In contrast, the amplitude map, $|E_z|$, which lacks such interference, does not match the experimental observations. Similar comparison methods have been employed in many previously reported studies [*Nat. Nanotechnol. 17, 940 – 946 (2022)*][*Nature 582, 209 – 213 (2020)*][*Nature 596, 362 – 366 (2021)*][*eLight 3, 14 (2023)*]. A comparison is commonly made between the simulated $Re(E_z)$ and the near-field amplitude, as presented in Figure R9.
Otherwise, the Fourier transform of our imaging data, limited by the sample's small size, is currently of insufficient quality for overlaying onto the iso-frequency contours in Figures 3c, d.

[*REDACTED*]

Figure R9. Experimental launching of polaritons and corresponding simulations in previous literatures. a, b, Schematic of the near-field polariton-launching experiment (a) and experimental near-field amplitude $|E_{in} + E_p|$ and $|E_p|$ (b), which are taken from Fig. 3 of the paper Hu et al. [*eLight 3, 14 (2023)*]. **c, d,** Experimentally measured near-field images (c) of polaritons and the corresponding numerically simulated near-field distributions (Re(Ez)) (d), which are taken from Fig. 2 of the paper Zheng et al. [*Science advances, 2019, 5(5): eaav8690*]. **e, f,** Experimentally measured near-field images (e) of polaritons and the corresponding numerically simulated near-field distributions (Re(Ez)) (f), which are taken from Fig. 3 of the paper Hu et al. [*Nat. Nanotechnol. 17, 940 – 946 (2022)*]

11. The authors state: "In this study, we focus exclusively on the analysis of polaritons at frequencies around the type-II band." To strengthen this discussion, they should include numerical simulations showing the spatial distributions of Re(Ez) across three distinct regions: i) the frequency range where CNT plasmons does not intersect with h-BN phonon polaritons; ii) the frequency range where CNT plasmons intersect with h-BN type I RB phonon polaritons; and iii) the frequency range where CNT plasmons intersect with h-BN type II RB phonon polaritons. What do the authors expect to observe in s-SNOM nano-imaging for type I RB frequencies in this system?

Our reply: We appreciate the reviewer's helpful comments and insightful suggestions.

The reviewer correctly highlights the significance of examining hBN's two Reststrahlen bands. While we considered both type I and type II hyperbolic phonon polaritons (HPPs) in our theoretical analysis, our experimental measurements were limited to type II HPPs. This limitation is due to the unavailability of continuous-wave laser sources operating in the 760–820 cm^{-1} range, which corresponds to type I HPPs. Furthermore, the anisotropic nature of hBN causes the out-of-plane mode (type I HPPs) to be significantly weaker than the in-plane mode (type II HPPs). Consequently, our experimental study primarily focused on the type II Reststrahlen band, which is also the most examined frequency range in hBN phonon polariton research.

To address the reviewer's request, we performed finite-element simulations using COMSOL to simulate the hybrid polariton mode in the type I HPPs of hBN, and the corresponding results are presented in Figure R10. It is noted that due to the emergence of well-defined hyperbolic phonon polaritons in hBN, the hyperbolic plasmons of the carbon nanotube array have also evolved into elliptical wavefront shapes.

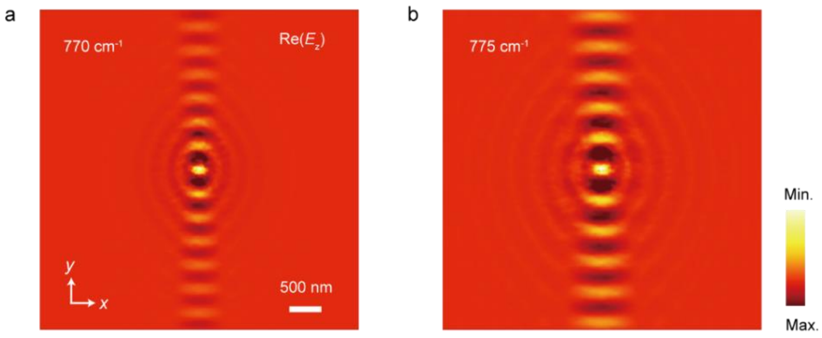

Figure R10. Numerical simulations of the hybrid polariton mode in the type I HPPs of hBN. The numerical simulated spatial distribution of the polariton field Re(E_z) measured above the CNT-array/hBN at frequencies

770cm^{-1} (a) and 775cm^{-1} (b), respectively.

12. Are the authors able to grow or transfer the CNT array onto different substrates (e.g. BaF_2 or SiO_2) and perform nano-imaging measurements there? I believe it would be highly beneficial for the manuscript to show a direct experimental and numerical comparison between the coupled system (CNT arrays/h-BN) and its individual constituents (CNT array on one side, h-BN on the other) including dispersion plots, Re(Ez) simulations, and s-SNOM measurements.

Our reply: We thank the reviewer for his/her important suggestions.

We have tried to transfer the CNT array onto different substrate, such as SiO_2; however, current dry transfer techniques disrupt the ordered, densely packed alignment of the nanotube array during the process.

According to the reviewer's suggestion, we have calculated the dispersion relation of the individual CNT array (without hBN) and included it in the Supplementary Information. Unfortunately, as the CNT array cannot be effectively transferred to a freestanding condition, we are currently unable to obtain experimental data for the dispersion of the isolated CNT array at this time.

13. Regarding the sample fabrication process, the authors could clarify whether CNT arrays could be integrated with other 2D van der Waals materials such as graphene, α-MoO_3 or α-V_2O_5.

Our reply: We thank the reviewer for his/her constructive suggestions regarding sample fabrication.

In our experiments, we have also attempted to integrate the CNT array with other two-dimensional materials (graphene, α-MoO_3, or α-V_2O_5) through stacking. As mentioned above, current transfer techniques tend to disrupt the structure of the CNT array during the process. However, we can transfer other two-dimensional materials successfully onto the CNT array, which is part of our future research plan.

14. The coupling strength and dispersion modification in plasmon–phonon systems are known to depend sensitively on the hBN thickness. The authors mention using a 55 nm-thick flake "to reduce phonon intensity," but no data or criteria are shown to justify this choice or demonstrate systematic thickness dependence. I believe that presenting simulations or experiments across several hBN thicknesses would greatly strengthen the coupling interpretation.

Our reply: We appreciate the reviewer's insightful suggestions regarding the dependence of coupling strength and dispersion on hBN thickness.

Indeed, the intensity and wavelength of phonon polaritons in hBN are thickness-dependent, as

reported in previous literature [*Science 343, 1125-1129 (2014)*]. As indicated in Fig. R11, thicker hBN leads to larger phonon polariton amplitude and longer polariton wavelength. In Fig. R12 below, we simulate the coupling between polaritons in hBN flakes of varying thicknesses and CNT array plasmons at a frequency of 1538 cm^{-1}. When the hBN flake is too thin, the resulting phonon polaritons exhibit an extremely small amplitude and a very short wavelength. This prevents effective coupling with plasmons in the CNT array, and the wavefront maintains its hyperbolic character. A transition to an elliptical wavefront occurs only when the hBN thickness is sufficient.

On the other hand, since hBN serves as a substrate material, its phonon response acts as a background signal. To prevent this phonon response from overwhelming the CNT array plasmon signal, we cannot use excessively thick hBN. Besides, as mentioned before, the wavelength of phonon polaritons in hBN is proportional to its thickness. Due to the size constraints of our CNT array sample, overly thick hBN flakes produce hybrid polaritons with a wavelength that exceeds the CNT array dimensions, thereby preventing the visualization of interference fringes. To achieve coupling visualization between phonon polaritons and plasmons, their wavelengths need to be comparable.

Taking all factors into consideration, we selected hBN with a thickness of several tens of nanometers. The 55 nm thickness is not necessarily optimal; rather, it represents a suitable choice that balances these conditions.

[*REDACTED*]

Figure R11. The evolution of the phonon polariton amplitude and wavelength with the thickness of hBN crystals. These figures are taken from Fig. 3 of the paper Dai et al. [*Science 343, 1125-1129 (2014)*]

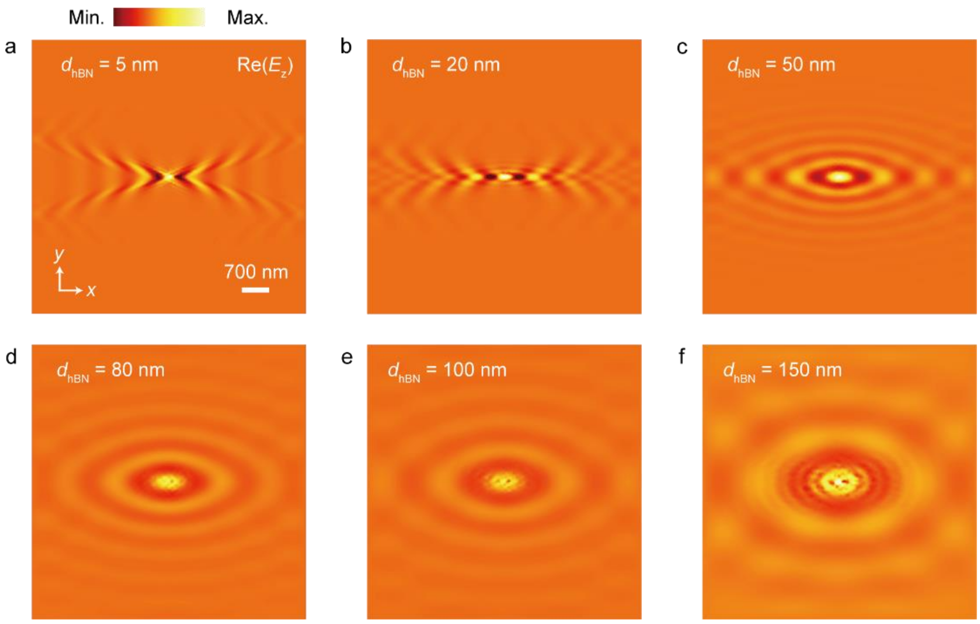

Figure R12. The hBN thickness dependence of the hybrid polaritons. a-f, The numerical simulated spatial distribution of the polariton field Re(Ez) measured above the CNT-array/hBN at the frequency of 1538cm^{-1} for hBN thickness of 5, 20, 50, 80, 100, and 150 nm, respectively.

15. As pointed out in previous comments, the comparison between experiment and simulation throughout the work is mostly qualitative ("good agreement"). The authors should provide quantitative comparisons to substantiate the agreement. Also, regarding the experimental results, several statements refer to "low loss" "long lifetime", and "high-quality factor" without quantitative support. Extracted propagation lengths or damping constants from s-SNOM data would be welcome and allow comparison with other polaritonic materials.

Our reply: We appreciate the reviewer's valuable suggestions regarding quantitative comparisons and the characterization of the experimental results.

For the comparison between experiment and simulation, a key quantifiable parameter is the wavelength. We provide a comprehensive comparison of wavelengths between experiment, calculations, and numerical simulations in Fig. 2e and Supplementary Figure 3.

As for the quality factor, we analyzed it using the definitions $Q = Re(q)/Im(q)$, and fitted the experimental near-field profile with the formula below:

$$I = \left|\eta_s + \eta_R \frac{1}{\sqrt{2x}} e^{\frac{-2x}{a}} e^{i(2qx+\phi_R)}\right|$$

where η_s is the scattering rate of incident light back into the detector, η_R is the reflectivity of HPs at the edge, q is the plasmon wavevector, a is the decay length, and ϕ_R is the reflection phase. This formula is derived from previous literature. [*Adv. Funct. Mater. 2019, 29, 1904662.*]

We selected the experimental data at frequencies of 1111 cm^{-1} and 1639 cm^{-1} for fitting, and the results are shown in Fig. R13 below. It should be noted that the quality factor of the CNT array

plasmons can reach as high as 21, and the compression factor is 21.57. The hyperbolic plasmons in our CNT array demonstrate a quality factor comparable to the widely studied systems of $MoOCl_2$ hyperbolic plasmons and α-MoO_3 hyperbolic polaritons [*Science387,786-791(2025)*] [*Nature 562, 557–562 (2018)*]. More importantly, our closed-loop CNT-array ring supports polaritons with a significantly enhanced quality factor of 67.63, as discussed in the subsequent question. This performance justifies our use of the term "low-loss" to characterize the system.

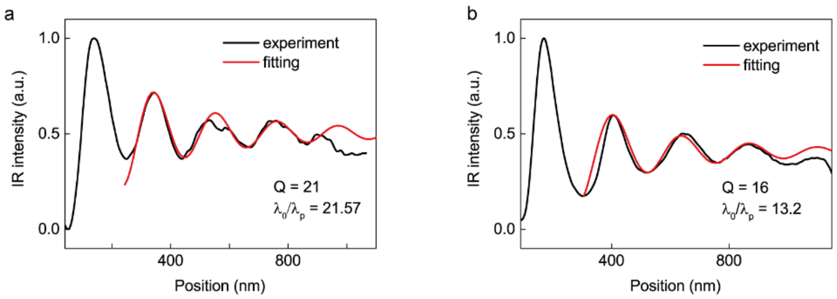

Figure R13. Extraction of the quality factor of plasmons in CNT array. a, The near-field profile along the axial direction of CNT array at the frequency of 1111 cm^{-1} and the corresponding fitting curve. **b,** The near-field profile along the axial direction of CNT array at the frequency of 1639 cm^{-1} and the corresponding fitting curve.

16. The interpretation of the observed near-field patterns in Figure 4 as whispering-gallery modes remains highly speculative. The near-field images presented in Figure 4 and the Supplementary Information are consistent with frequency-dependent interference effects, but they do not constitute conclusive evidence of resonant mode confinement. To substantiate the claim of whispering-gallery plasmonic modes, the authors should (i) perform full-wave electromagnetic simulations of the circular CNT-array/hBN structure to visualize the modal field distributions and confirm the existence of azimuthally confined resonances; (ii) provide frequency-dependent spectral data from s-SNOM amplitude or phase measurements to identify discrete resonance peaks; and (iii) estimate the corresponding quality factors (Q) to demonstrate true cavity behavior. Additionally, a systematic comparison across rings of different diameters would enable verification of the expected quantized angular momentum dependence (mode number scaling with circumference). Without such quantitative analysis and modeling, the evidence remains insufficient to support the claim of an "on-chip whispering-gallery nanocavity" and the observed oscillations could alternatively arise from simple standing-wave interference in the hybrid polariton field.

Our reply: We appreciate the reviewer's significant suggestions and have addressed each comment point by point, as outlined below.

(i) We have performed numerical simulations of polariton whispering gallery modes (WGMs) and incorporated the results into Figure 4 of the revised manuscript, specifically shown as Figure R14. Notably, the simulation results are in excellent agreement with the experimental observations, confirming that the signals observed in the experiment indeed originate from WGMs. Regarding the mode field distribution: the CNT arrays we fabricated exhibit an almost ideal circular geometry, enabling the polaritons to propagate along the circumference in a WGM-like manner. Due to the

absence of reflective boundaries that would generate noticeable interference patterns, it is challenging to directly obtain a clear angular mode field distribution in the experiment. Consequently, when the atomic force microscope (AFM) probe is positioned along a ring of the same diameter, the conditions for near-field excitation and signal collection remain unchanged. This results in a nearly uniform infrared intensity within the same-diameter ring in nano-infrared imaging. The same principle applies to rings with different diameters. In the corresponding numerical simulations, this scenario is modeled by scanning the position of an electric dipole to emulate the tip-scanning process, while simultaneously recording the electric field E_z component directly beneath the dipole.

(ii)-(iii) We have collected the frequency-dependent near-field signal intensity, with the background subtracted at a certain point (denoted by a blue dot in Fig. R15a) on the closed-loop CNT-array ring on hBN, and the results are plotted in Figure R15b. The data are well fitted by a standard resonance peak curve, confirming that the observed signal corresponds to a whispering-gallery mode. From this fitting, the quality factor (Q) is extracted as follows: $Q = \frac{\omega_c}{\Delta\omega} = \frac{1600.16\ cm^{-1}}{23.66\ cm^{-1}} = 67.63$, where ω_c is the resonance frequency and $\Delta\omega$ is the full width at half maximum (FWHM) of the resonance peak.

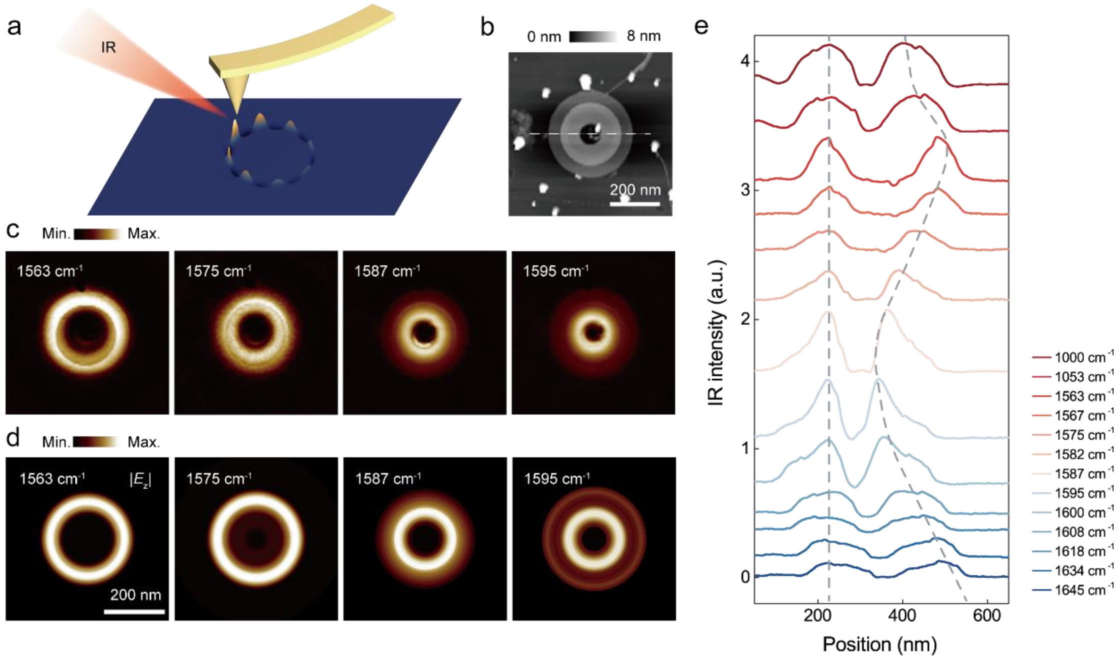

Figure R14 The revised version of Figure 4 of the manuscript. a, Schematic of infrared nano-imaging of whispering-gallery polaritonic mode in the CNT-array/hBN by using s-SNOM. **b,** AFM topography image of a closed-loop CNT array ring on hBN. **c, d,** Infrared nano-imaging and corresponding numerical simulations of polaritons in the CNT-array/hBN at different frequencies, respectively. **d,** The Near-field line profiles along the sections marked by the dashed line in (b).

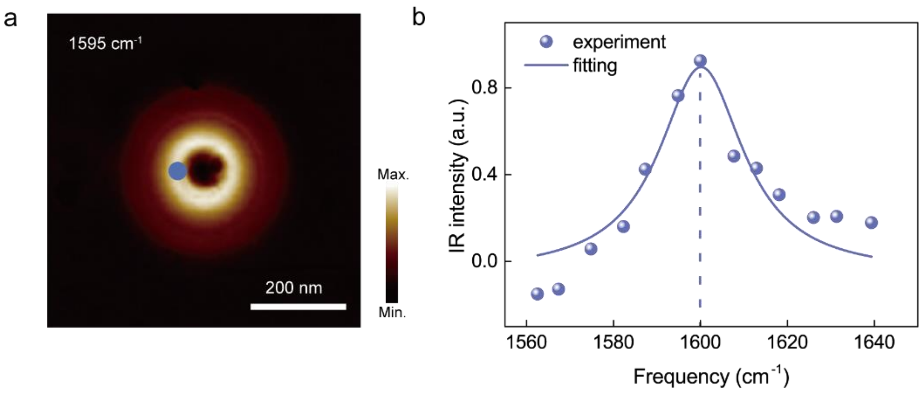

Figure R15 Extraction of the quality factor of the whispery-gallery mode polaritons in closed-loop CNT array/hBN. **a,** A typical infrared nano-imaging of polaritons in the CNT-array/hBN. **b,** Frequency-dependent near-field signal intensity at a certain point on the closed-loop CNT-array ring on hBN. The position where the experimental data were extracted from is denoted by a blue dot in (a).

17. The Discussion section includes numerous overstatements ("approached physical limits", "unprecedented capabilities", "lays a solid foundation"). These claims should be revised or supported by quantitative metrics.

Our reply: We appreciate the reviewer's valuable suggestions.

In response, we have removed all the aforementioned overstatements and made comprehensive revisions to the manuscript. The specific changes are as follows:

a. Revised "This on-chip natural whispering-gallery nanocavity structure has approached the physical limits, with its diameter reduced to about a hundred nanometers" to "This on-chip natural whispering-gallery nanocavity structure features a diameter reduced to approximately one hundred nanometers".
b. Revised "showcasing unprecedented capabilities for on-chip manipulation of whispering-gallery optical fields" to "exhibiting capabilities for on-chip manipulation of whispering-gallery optical fields".
c. Revised "laying a solid foundation for the realization of integrated optoelectronic systems" to "providing a new pathway for achieving integrated optoelectronic systems".

Response to Reviewer #3

The manuscript reports configurable plasmon – polaritons arising from the coupling between hyperbolic plasmons and phonon polaritons in CNT array/hBN heterostructures. Infrared (IR) nano-imaging is performed as a function of excitation laser energy, which reveals multiple branches of coupled phonon plasmon modes. Topological transition of a hyperbolic isofrequency contour to an elliptical one is observed using IR nano-imaging and from numerical calculations. Plasmonic whispering-gallery modes are presented in closed-loop CNT array ring on a hBN substrate. Due to the lack of novelty and impact of the work, and multiple significant claims with insufficient evidence and premature data analysis, the reviewer does not recommend the publication of this manuscript in Nature Communications.

Our reply: We appreciate the reviewer's evaluation of our work and the valuable feedback provided. Below, we address each question and comment raised by the reviewer one by one.

Comment 1: First and foremost, the main concern of the manuscript is the lack of novelty. Hyperbolic polaritons have been demonstrated numerous times in other vdW materials, with canonical examples including phonon polaritons hBN and a-MoO3 as the authors have referenced as well as in nanopatterned hBN [Science 359, 892 (2018)]. Hyperbolic plasmons have also been demonstrated in multiple other vdW materials, including in ZrSiSe at infrared frequencies [Sci Adv 8, add6169 (2022)] and in MoOCl2 at visible frequencies [Science 387, 786 (2025)]. The properties claimed by the authors (deep subwavelength confinement, broad spectral range, etc) are generic to these known (plasmonic) hyperbolic systems. Furthermore, the coupling-induced topological transition of polaritons has been repeatedly demonstrated in other vdW materials platforms, including in twisted a-MoO3 [Nature Mater 19, 1307 (2020); Nature 582, 209 (2020)] or in graphene/a-MoO3 [Nature Nanotech 17, 940 (2022); Nature Commun 13, 3719 (2022)].
Therefore, the reviewer does not find that the work provides a sufficient conceptual advance to warrant publication in Nature Communications.

Our reply: We sincerely thank the reviewers for their thorough and systematic review of work in this field.

We fully agree that the concept of "hyperbolic polaritons and coupling-induced topological transitions" has been extensively studied in van der Waals materials. Therefore, the core innovation of this work does not lie in merely reproducing this phenomenon, but rather in the construction and validation of a novel platform with fundamentally different properties and unique advantages for manipulation: by utilizing directly grown, ultra-clean CNT arrays as a natural hyperbolic polariton substrate, we have achieved broadband, low-loss, and directly imageable in-plane hyperbolic plasmons, as well as tunable topological transitions through hybridization with hBN phonon polaritons. The distinctiveness of this platform is demonstrated in the following four

key aspects:

(1) Broadband In-Plane Hyperbolic Plasmons vs. Narrowband Phonon Hyperbolic Polaritons
The hBN, α-MoO_3, and other systems mentioned by the reviewers primarily rely on phonon polaritons, whose hyperbolic response is constrained by the Reststrahlen band, resulting in inherently narrow bandwidths. In contrast, our work leverages the effective medium anisotropy of CNT arrays, where the hyperbolic behavior originates from the Luttinger-liquid plasmon properties of CNTs. This enables high Q-factors and nearly linear dispersion over an exceptionally broad spectral range (e.g., 6–11 μm). Our platform fundamentally differs from conventional narrowband phonon systems in terms of bandwidth, loss levels, and mode controllability.

(2) In-Plane Wavefront Visualization and Manipulation vs. Out-of-Plane Propagation Limitations
Unlike the out-of-plane propagation or vertically quantized modes commonly observed in systems such as ZrSiSe, our CNT array platform supports in-plane hyperbolic plasmons. This allows for direct imaging and observation of isofrequency profiles and energy flow directions, overcoming the geometric constraints of vertical propagation in atomically thin layers. Consequently, it enables practical, on-demand manipulation of in-plane momentum propagation and wavefront shapes, offering a new level of control.

(3) Ultra-Clean Direct Growth vs. Nanofabrication or Twisting Engineering
While nanopatterning techniques (e.g., etching) can induce hyperbolicity, they often introduce edge roughness and defect scattering, which degrade performance. In contrast, our platform employs directly grown, crystal-scale CNT arrays with atomically smooth surfaces and minimal impurity scattering. This "ultra-clean" approach eliminates the need for complex nanofabrication or twisting engineering, thereby directly reducing polariton transmission losses and significantly enhancing experimental reproducibility.

(4) Universal Coupling Platform vs. Strict Frequency Matching
Regarding coupling-induced topological transitions, our work goes beyond merely replicating existing concepts (e.g., twisted α-MoO_3) by extending them to a broadband hyperbolic plasmonic platform. The broadband hyperbolic response of CNT arrays significantly relaxes the frequency-matching requirements for coupled systems, functioning as a universal "coupling platform" that allows greater design flexibility for hybridization with various polariton modes (e.g., phonons, plasmons) without the stringent material and frequency constraints of narrowband systems.

In conclusion, the innovation of this work lies in the establishment of an ultra-clean, low-loss, broadband, and directly imageable in-plane hyperbolic plasmon platform. This provides a scalable and practical pathway for polariton manipulation and topological transitions without relying on complex fabrication techniques. Our work represents a substantial advancement in the development of polariton-based devices and tunable nanophotonics, far beyond merely reproducing known phenomena in new materials.

Furthermore, we have incorporated the references suggested by the reviewer (*Science 359, 892 (2018); Sci Adv 8, add6169 (2022); Science 387, 786 (2025); Nature Mater 19, 1307 (2020); Nature*

582, 209 (2020); Nature Nanotech 17, 940 (2022); Nature Commun 13, 3719 (2022)) as citations in the revised manuscript.

Comment 2: Another significant concern is that the manuscript repeatedly makes claims that are not substantiated by sufficient experimental evidence or logical derivation, which compromises the scientific integrity of the manuscript.

2-i: The claim of "configurability" based on simple frequency dependence is misleading. This is a basic material property of CNT array/hBN device, but not an externally tunable feature that "configurability" typically implies. The authors' method of labelling "configurability" can be applied to most all vdW polaritonic materials. For example, phonon polaritons in hBN exist only within the Reststrahlen band. In this case, hBN phonon polaritons would also be "configurable" as one can switch phonon polaritons on and off simply by tuning frequency.

Our reply: We thank the reviewer for his/her insightful comments

To prevent misunderstanding, we have revised all mentions of "configurable polaritons" in the text to "frequency-dependent topological polaritons," and changed the title to " Frequency-dependent topological polaritons in carbon nanotube array/hBN heterostructures". The polaritons reported in this work exhibit distinct topological characteristics (hyperbolic or elliptical) depending on frequency, which better captures the fundamental nature of this system.

We demonstrated the unique capability of our polaritons to tune their wavefront shape and wavelength simply by adjusting the excitation frequency across a wide spectral range, an unprecedented feature that distinguishes our system from other conventional ones. Our approach offers an ultra-clean, efficient, and versatile solution for on-demand polariton manipulation, completely eliminating the need for complicated twist-angle engineering in van der Waals heterostructures or complex micro/nanofabrication of electrodes for electrostatic gating. Therefore, this inherent advantage of our system enables direct optical control without physical modifications or additional fabrication steps.

2-ii: The authors claim to be the "first" to demonstrate, "a whispering-gallery plasmonic mode confined in closed-loop CNT array on hBN." However, this claim of novelty is overstated. As the authors' own citations (Ref. 38) confirm, whispering-gallery plasmonic modes have already been demonstrated in other vdW materials, such as graphene. Therefore, the simple extension of this work to a CNT array does not seem to be a significant conceptual advance, and the manuscript fails to convey the importance of this observation in CNT arrays.

Our reply: We thank the reviewer for his/her constructive suggestions.

We refer to being the "first" to demonstrate a whispering-gallery plasmonic mode *confined in CNT arrays*. Due to the extremely small diameter of CNTs, typically around 1 nm, it is experimentally

difficult to observe whispering gallery modes within their cross-sections (such as the polariton whispering gallery modes observed in BN nanotubes [*Nat. Nanotechnol. 18, 529–534 (2023)*]). Therefore, forming CNTs into nano-rings is essential to achieve this phenomenon. We are the first to assemble CNTs into close-loop nano-rings, and have observed a plasmonic whispering gallery mode with a very high quality-factor (as described in the following Fig. R18) and excellent coherence.

To prevent any misunderstanding, we have removed the word "first" from the revised manuscript.

2-iii: In addition, the authors' claim of a whispering-gallery plasmonic mode itself is not convincing. Plasmonic whispering gallery modes are those that travel around a circumference of the cavity. Based on the main dataset shown in Fig. 4c, the reviewer does not see any clear signatures of plasmonic wave travel around the circular, donut shaped structure, forming a whispering-gallery mode. IR nano-imaging shown in Fig. 4c seems rather a result of tip-launched (or cavity edge launched) plasmons interacting with the entire cavity structure, forming a cavity-like nano-IR response, but not necessarily a whispering-gallery mode specific to the chiral motion of polaritons around the loop. More rigorous analysis needs to be performed to support their claim of whispering-gallery modes.

Our reply: We appreciate the reviewer's valuable feedback regarding the interpretation of the observed phenomena as whispering-gallery plasmonic modes.

The polaritons we observe are excited by an AFM tip and propagate along the axial direction of carbon nanotubes, completing a full loop before being detected again by the tip. Since our ring-shaped CNT array is highly centrosymmetric and perfectly circular, when the tip is positioned above a path at the same radius, the excitation of polaritons is identical, and the detected signals are also identical. This is manifested as completely identical infrared signals along the same radial path in the final near-field imaging.

However, paths at different radii display varying brightness—either brighter or darker—corresponding to the constructive or destructive interference of the polaritons. Notably, this brightness contrast changes with the excitation frequency, which we attribute to the presence of the whispering-gallery mode of polaritons.

To further support our claim that these are indeed whispering-gallery modes propagating along the axial direction of the CNT array—as opposed to radial propagation modes— we have selected a larger closed-loop CNT array ring for analysis. The results, presented in Fig. R16, illustrate that the path length for polaritons to complete one loop (i.e., the circumference of the circle) is significantly longer, far exceeding the propagation distance of the polaritons. As a result, no constructive or destructive interferences are observed in this configuration.

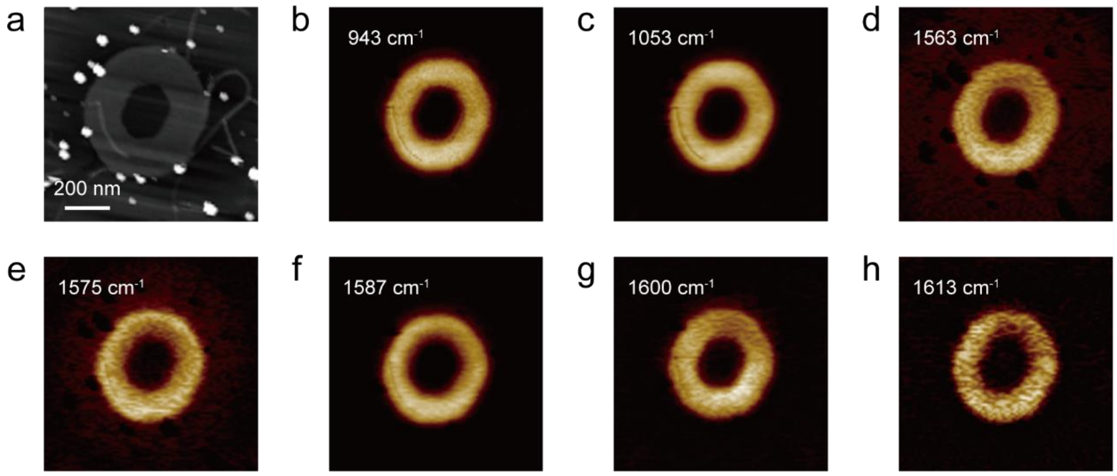

Figure R16. The evolution of the hybrid polaritons in a larger closed-loop CNT array ring on hBN with the excitation frequency, displaying nearly no interference. a, AFM topography image of a closed-loop CNT array ring on hBN. b-h, Infrared nano-imaging of plasmons in the CNT-array/hBN at different frequencies.

2-iv: The authors claim that "the synergy of the low-loss characteristics of the Luttinger liquid electronic state, uniform chirality, and near-atomic-level smooth, lossless ring-shaped interfaces enables efficient optical field confinement and the formation of strong optical resonances," is highly speculative and lacks quantitative support. The manuscript provides no metrics to define, measure, and quantify the efficient optical field confinement and strong optical resonances. Furthermore, the authors do not demonstrate how their data supports the existence of or the connection to the low-loss Luttinger liquid, uniform chirality, or lossless interfaces. These claims are concerning because they are presented without any supporting evidence. At the very least, the authors should provide a quantitative comparison of the polariton quality factors in their straight and circular structures.

Our reply: We thank the reviewer for his/her comments.

The plasmonic properties of carbon nanotubes have been reported to exhibit high spatial confinement capability and high quality factors in previous literatures. [*Nature Photon. 9, 515-519 (2015)*]

We performed a quantitative analysis of the Q factors of polaritons in the CNT array/hBN system, using the definitions of the quality factor $Q = Re(q)/Im(q)$ and the compression factor $N = Re(q)/k_0$, and fit the experimental near-field profile with the formula below:

$$I = \left| \eta_s + \eta_R \frac{1}{\sqrt{2x}} e^{\frac{-2x}{a}} e^{i(2qx+\phi_R)} \right|$$

where η_s is the scattering rate of incident light back into the detector, η_R is the reflectivity of HPs at the edge, q is the plasmon wavevector, a is the decay length, and ϕ_R is the reflection phase. This formula is derived from the previous literature [*Adv. Funct. Mater. 2019, 29, 1904662*]. We selected experimental data at frequencies of 1111 cm^{-1} and 1639 cm^{-1} for fitting, and the results are shown in Fig. R17 below. It can be observed that the Q factors of the polaritons in the CNT

array/hBN exceed 20, with a wavelength compression ratio reaching 21.

Additionally, we collected the near-field signal intensity with the background subtracted, at a certain point (denoted by a blue dot in Fig. R18a) on the closed-loop CNT-array ring at different excitation frequencies and the results are plotted in the Fig. R18b. The data are well fitted by a standard resonance peak curve. From this fitting, the quality factor (Q factor) is extracted as follows: $Q=\frac{\omega_c}{\Delta\omega}=\frac{1600.16\ cm^{-1}}{23.66\ cm^{-1}}=67.63$, where ω_c is the resonance frequency and $\Delta\omega$ is the full width at half maximum (FWHM) of the resonance peak.

These results demonstrate the advantage of achieving both a relatively strong spatial confinement and a high Q factor compared to other two-dimensional metallic plasmonic systems. Typically, strong spatial confinement can only be achieved at the expense of a low Q factor due to the absorption loss of metals. For instance, a two-dimensional surface plasmon at a silver/air interface has a quality factor of ~16 with $\lambda_p/\lambda_0 = 1.16$ at 400 nm, but Q worsens to ~2.27 when λ_p/λ_0 increases to 1.51 at 350 nm. [*Nature Photon. 9, 515-519 (2015)*] However, due to the different scattering mechanisms between plasmons and phonon polaritons, the Q factors of our coupled polaritons are lower than those of phonon polaritons in systems like hBN, which is inherently attributed to electron-electron scattering in plasmons.

In summary, compared to two-dimensional metallic plasmons, the polaritons we report exhibit advantages in wavefront tunability and higher Q factors. In contrast to hyperbolic phonon polaritons in materials such as α-MoO_3, our coupled polaritons offer advantages in broadband response and wavefront tunability.

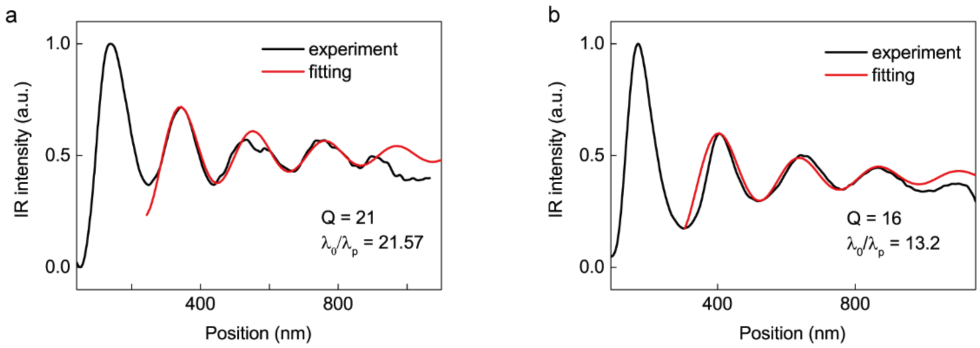

Figure R17. Extraction of the quality factor of plasmons in CNT array. a, The near-field profile along the axial direction of CNT array at the frequency of 1111 cm^{-1} and the corresponding fitting curve. **b,** The near-field profile along the axial direction of CNT array at the frequency of 1639 cm^{-1} and the corresponding fitting curve.

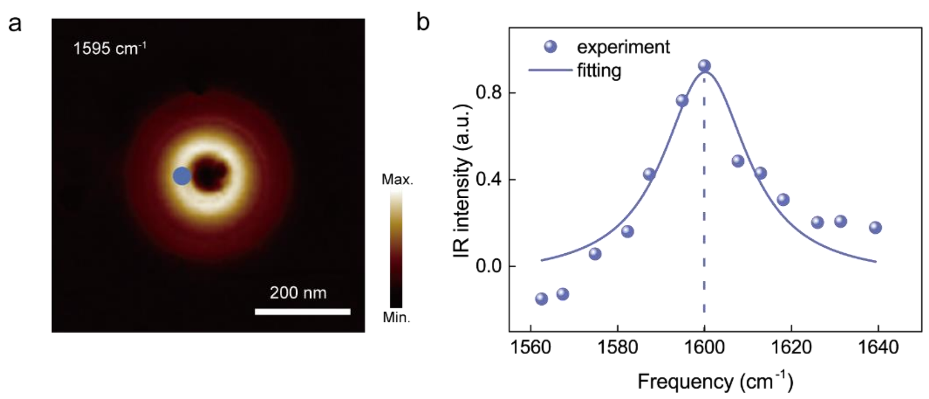

Figure R18. Extraction of the quality factor of the whispery-gallery mode polaritons in closed-loop CNT array/hBN. a, A typical infrared nano-imaging of polaritons in the CNT-array/hBN. **b,** Frequency-dependent near-field signal intensity at a certain point on the closed-loop CNT-array ring on hBN. The position where the experimental data were extracted from is denoted by a blue dot in (a).

2-iv: The authors also make a direct comparison of their whispering gallery modes with that in Ref. 38, stating that "characteristic dimensions and performance have improved by nearly an order of magnitude" in their materials with "high compression ratio." However, this comparison to Ref. 38 is misleading. Dimensions may be smaller than the ones shown in Ref. 38, but it is unclear how the performance is improved. The authors' claim that their compression ratio is higher, but this is not supported quantitatively. The compression ratio (or perhaps the effective mode volume) of polaritons does not have one-to-one correspondence with the physical dimension of the cavity. While physical dimensions can be smaller, effective mode volume can be larger. One needs to perform a rigorous quantitative analysis to make a case for such a claim. Without a quantitative analysis, the authors' claim is unsubstantiated.

Our reply: We thank the reviewer for his/her significant comments.

The term "characteristic dimensions and performance" in the manuscript refers specifically to our mode volume ratio V_p/V_0, which we estimate to be one order of magnitude lower than that of the nanocavity reported in Reference 38 [*Nat. Mater. 22, 838–843 (2023)*]. This estimation method follows the approach in previous literatures [*Science 360, 291-295 (2018)*] [*Science 368, 1219-1223 (2020)*], where the polariton wavelength $\lambda_p = \lambda_0/16.5$ is used. Given that the thickness of our nanocavity is on the atomic scale—approximately 4 nm—we obtain $V_p/V_0 = 2.3 \times 10^{-6}$. This value is nearly an order of magnitude smaller than the previously reported value of 10^{-5} in Reference 38 [*Nat. Mater. 22, 838–843 (2023)*].

Other minor comments: -hBN phonon polaritons are also hyperbolic. Can the authors comments on if the hyperbolicity of phonon polaritons play any role in the coupling and topological transition of hyperbolic plasmons in CNT array/hBN system? -The authors selected an hBN flake with a thickness of 55 nm to demonstrate the polaritonic coupling. Why does this particular thickness allow the intensity of the hBN phonon-polaritons to match that of the CNT plasmons? Do the authors have

any data or calculations to justify that 55 nm is the optimal thickness?

Our reply: We appreciate the reviewer's interesting suggestions.

As previously noted, although hBN supports out-of-plane hyperbolic phonon polaritons, these do not directly govern the coupling and topological transitions of in-plane hyperbolic plasmons in the CNT array/hBN system. Specifically, within Reststrahlen band 2, hBN exhibits in-plane isotropy and out-of-plane hyperbolic anisotropy. Due to finite thickness effects, the out-of-plane hyperbolic dispersion of hBN is quantized, which in turn modulates the size of the in-plane circular isofrequency contours. Consequently, it is not the out-of-plane hyperbolic dispersion itself that directly participates in the hybrid coupling within the CNT array/hBN heterostructure; rather, it is the in-plane isofrequency contours—tuned by the hBN thickness—that determine the momentum-matching conditions with CNT plasmons.

We chose this specific thickness of hBN because previous studies have shown that the strength and wavelength of hBN phonon polariton depends on thickness [*Science 343, 1125-1129 (2014)*]. As indicated in Fig. R19, the thicker hBN leads to the larger polariton amplitude and larger polariton wavelength. Numerical simulations for the coupling between polaritons in hBN of varying flake thicknesses and CNT array plasmons are shown in Fig. R20. If the hBN flake is too thin (for example, 5 nm thick), the hybrid polaritons maintain the hyperbolic character due to the extremely small amplitude and a very short wavelength of hBN phonon polaritons. A transition to an elliptical wavefront occurs only when the hBN thickness is sufficient.

Regarding excessively thick hBN flakes, several shortcomings emerge. First, since hBN functions as a substrate, its phonon response serves as a background signal. Overly thick hBN will mask the plasmon signal from the CNT array. Additionally, as previously noted, the wavelength of phonon polaritons in hBN scales with its thickness. Given the finite size of our CNT array sample, overly thick hBN flakes generate hybrid polaritons with wavelengths that exceed the spatial dimensions of the array, precluding the observation of interference fringes. To enable clear visualization of the coupling between phonon polaritons and plasmons, their wavelengths must be comparable.

Taking all factors into consideration, we selected an appropriately thin hBN flake with a thickness of several tens of nanometers—suitable for our purpose, though not necessarily the optimal choice.

Figure R19. The evolution of the phonon polariton amplitude and wavelength with the thickness of hBN crystals. These figures are taken from Fig. 3 of the paper Dai et al. [*Science 343, 1125-1129 (2014)*]

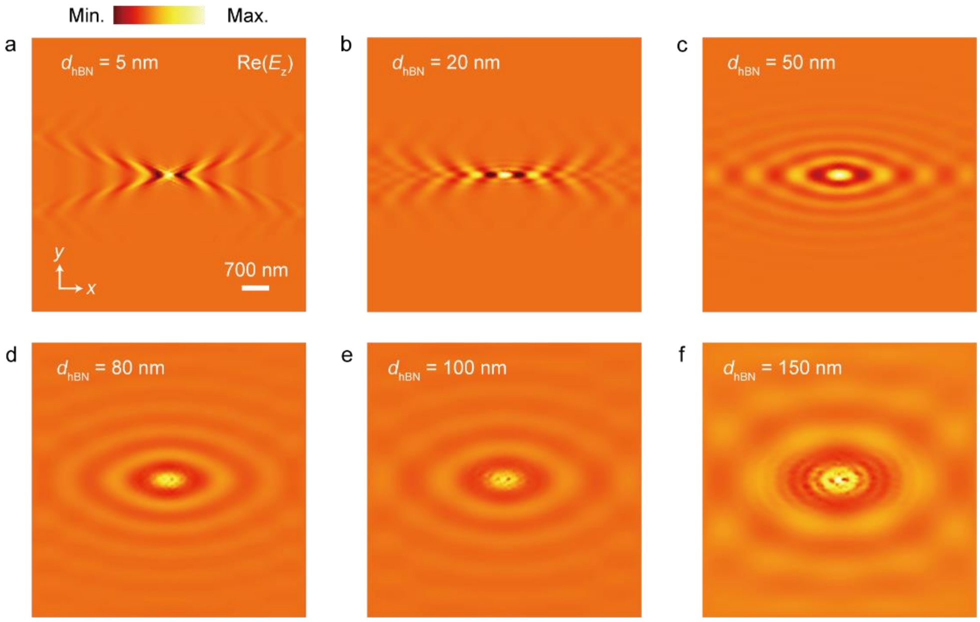

Figure R20. The hBN thickness dependence of the hybrid polaritons. a-f, The numerical simulated spatial distribution of the polariton field Re(Ez) measured above the CNT-array/hBN at the frequency of 1538cm^{-1} for hBN thickness of 5, 20, 50, 80, 100, and 150 nm, respectively.

Response to Reviewer #4

I co-reviewed this manuscript with one of the reviewers who provided the listed reports. This is part of the Nature Communications initiative to facilitate training in peer review and to provide appropriate recognition for Early Career Researchers who co-review manuscripts.

Our reply: We thank the referee for letting us know about his/her involvement in the Nature Communications initiative. It's commendable to see efforts towards training in peer review and recognizing the contributions of Early Career Researchers. The referee’s commitment to this initiative is valuable for the research community.

Response to Reviewers' Comments (NCOMMS-25-86882A)

We sincerely thank all reviewers for their careful evaluation of our work and for their insightful and constructive feedback. Below are our detailed point-by-point responses.

Response to Reviewer #1:

The authors addressed my comments and could resolve most issues. While the revision has improved and strengthened the manuscript and the manuscript contains novel and interesting results worth publishing, some inaccuracies remain. Particularly, the comments related to the whispering-gallery modes (previous comment 5) have not fully been resolved.
Please find some detailed comments below.

Our reply: We sincerely thank the reviewer for their positive evaluation and constructive feedback, which have significantly contributed to strengthening the manuscript. We acknowledge that some concerns remain, particularly regarding the whispering-gallery modes. Below we address each of the reviewer's questions and comments point by point.

1. The abstract now mentions "configurable plasmon frequency-dependent topological polaritons". I assume this does not indicate any topological protection but simply the transition between the open and closed-loop shape of the IFC, is this correct? If so, this could be clarified.

Our reply: We thank the reviewer for his/her insightful comments and suggestions.

The term "topological polaritons" in our manuscript indeed refers to the topological transition of the iso-frequency contours shape, rather than topological protection. This concept have been extensively studied and reported in the field of nanophotonics, as documented in the following references: [*Nature 582, 209 – 213 (2020)*][*Nat. Nanotechnol. 17, 940 – 946 (2022)*][*Nat. Mater. 19, 1307 – 1311 (2020)*][*Nat. Commun. 13, 3719 (2022)*]. To clarify this point, we have added the following explanation:

"*Here, with a homemade scattering-type scanning near-field optical microscope (s-SNOM), we experimentally demonstrate nanoscale frequency-dependent topological polaritons—specifically, the topological transition of polariton iso-frequency contours between open and closed-loop shapes, in CNT-array/hBN heterostructures.*"

2. The authors now mention "as the two interference peaks move closer together, the interference pattern becomes more strongly constructive". The authors could further clarify how "constructiveness" can be quantified/seen from Fig. 4c.

Our reply: We thank the reviewer for his/her insightful comments.

To clarify the concept of "constructiveness", we extracted the near-field intensity profile (reflecting the magnitude of the E_z component) from Fig. 4c and plotted it in Fig. 4e. The relationship between the peak-to-peak distance and the peak height in the profile curve of Fig. 4e is shown in the Fig. R1. It can be observed that a smaller peak-to-peak distance corresponds to a higher resonance peak, indicating a more strongly constructive interference pattern.

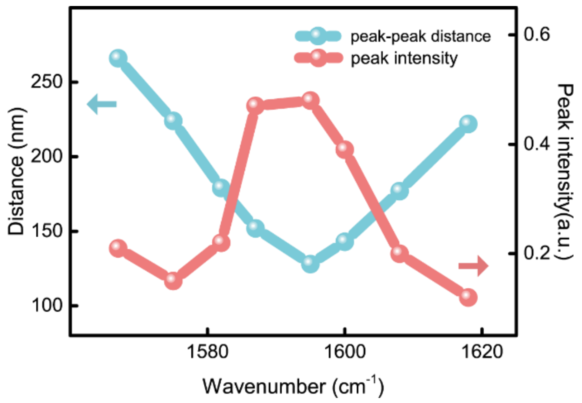

Figure R1. The relationship between the peak-to-peak distance and the peak height of the constructive interference peak. These data were extracted from the Fig.4e of the manuscript.

3. Regarding the whispering-gallery modes, the added comparison with the numerical simulations is helpful. However, some comments are still not sufficiently clear. In their response and revised supplementary information, the authors mention "The results (see Supplementary Fig. 9b), which exhibit a strong fit to a standard resonance peak curve, confirm the presence of a whispering-gallery mode." It is not clear to me why "a strong fit to a standard resonance peak curve" should indicate whispering-gallery mode polaritons. Rather a "standard" resonance peak curve should have various possible explanations, with whispering-gallery mode polaritons being only one of many. As such, the authors should clarify which parts of the results confirm the presence of a whispering-gallery mode.

Our reply: We thank the reviewer for his/her constructive comments.

A standard resonance peak curve indicates the presence of polariton interference resonance within the array, which we attribute to the whispering-gallery mode of polaritons. To better explain the whispering gallery mode, we performed further numerical simulations. The results are shown in the Fig. R2.

In these simulations, we maintained the radial width of the CNT array consistent with that of the experimental sample while appropriately increasing the axial length to observe more periods. To intuitively visualize the phase information of the polaritons, we placed an electric dipole above the array to excite the polaritons and extracted the Re(E_z) component from above the array. The simulation results clearly demonstrate the characteristics of whispering gallery modes of the polaritons. As the incident frequency increases, the polariton wavelength decreases, and the

number of interference nodes in the standing wave increases.

Moreover, these simulation results align with our experimentally observed tip-excite-and-tip-probe conditions, confirming that constructive interference manifests in circular patterns with varying perimeters, consistent with the behavior expected from whispering-gallery modes.

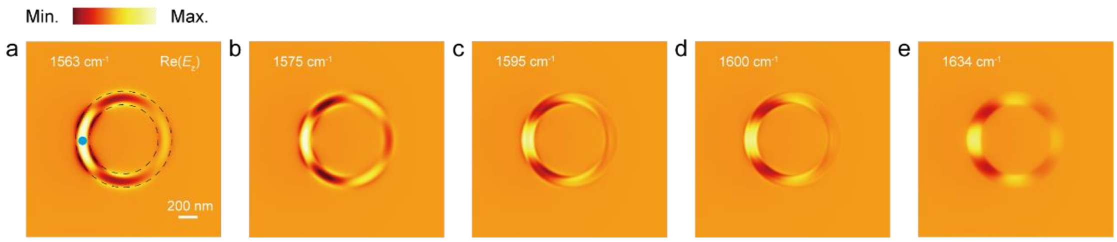

Figure R2. The evolution of whispering-gallery mode of polaritons in CNT-array/hBN at different frequencies. The excitation frequencies are 1563cm^{-1} (a), 1575cm^{-1} (b), 1595cm^{-1}(c), 1600 cm^{-1} (d) and 1634cm^{-1} (e), respectively. The blue dot in (a) represents the electric dipole for exciting the polaritons. The dashing black circles represents the boundary of the CNT array loop.

4. The claims on the whispering-gallery modes should generally be weakened, e.g., using weaker wording, or additional discussion should be included in the manuscript and/or supplementary information. For example, parts of the response to the different reviewers' comments might be included in the manuscript/SI instead of in the rebuttal letter only, e.g., Fig. R16 and corresponding discussion.

Our reply: We thank the reviewer for his/her constructive suggestions.

We have already incorporated Fig.R3 (Fig. R16 of the last rebuttal letter) into the revised Supplementary Information, and have added the following description in the revised SI.

"To further support our claim that these are indeed whispering-gallery modes propagating along the axial direction of the CNT array—instead of radial propagation modes— we have selected a larger closed-loop CNT array ring for analysis. The results, presented in Supplementary Fig. 10, illustrate that the path length for polaritons to complete one loop (i.e., the circumference of the circle) is significantly longer, far exceeding the propagation distance of the polaritons. As a result, no constructive or destructive interferences are observed in this configuration."

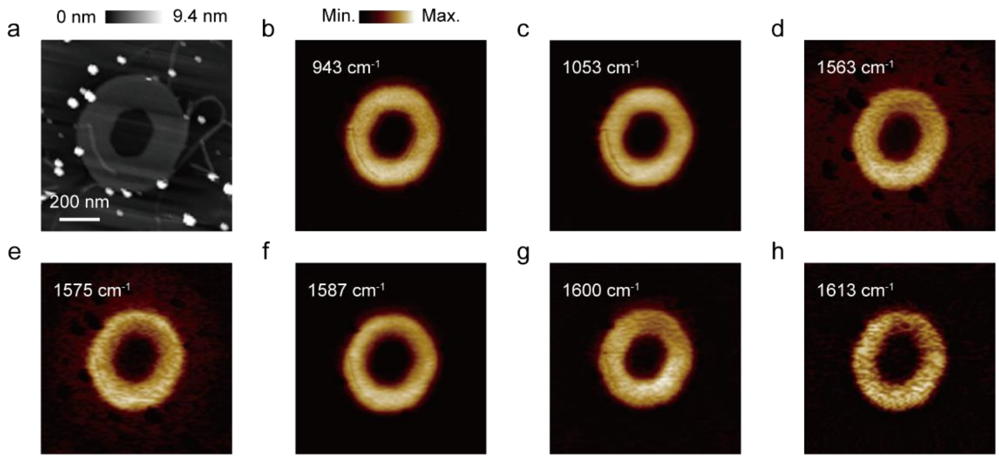

Figure R3. The evolution of the hybrid polaritons in a larger closed-loop CNT array ring on hBN with the excitation frequency, displaying nearly no interference. a, AFM topography image of a closed-loop CNT array ring on hBN. b-h, Infrared nano-imaging of plasmons in the CNT-array/hBN at different frequencies.

5. Finally, the authors still state that "Compared to traditional low-dimensional polaritonic whispering-gallery nanocavities, its characteristic dimensions and performance have improved by nearly an order of magnitude." In their rebuttal, the authors indicate that the claimed improvements mainly refer to the critical dimensions. However, the wording in the authors' manuscript still indicates a broader "performance" improvement by "nearly an order of magnitude". The authors should either clarify, in the manuscript, that the improvement solely refers to the mode volume (or even just to the physical dimension), or they should detail which other performance improvements they refer to compared to previous work.

Our reply: We thank the reviewer for his/her significant suggestions.

The reviewer is correct that the improvement solely refers to the mode volume. To clarify the comparison, we have revised the statement as follows,

"*Compared to traditional low-dimensional polaritonic whispering-gallery nanocavities, its characteristic dimensions have improved by nearly an order of magnitude. Its mode volume is as small as on the order of* 10^{-6}."

Response to Reviewer #2:

The authors have adequately addressed my comments. I recommend publication in its present form.

Our reply: We sincerely thank the reviewer for his/her appreciation of our work and recommendation for the publication of our manuscript.

Response to Reviewer #3:

The authors have made a significant improvement of the manuscript and clearly addressed most of my previous comments. Before the acceptance of the manuscript, I just a have a few minor points that need to be clarified and would further strengthen the manuscript.

Our reply: We sincerely thank the reviewer for his/her appreciation of our work and for the positive evaluation.

1) Q-factor of 60 derived from the frequency dependent measurements of a cavity is exceptionally high. Can the authors explain why such a high Q-factor is possible? This three-fold enhancement compared to Q-factors of 20 in straight devices derived by the authors is counterintuitive. Either the specific method of extracting Q-factor of 60 is inaccurate or there is an unknown procedure that enhances the Q-factor in a closed-loop CNT array versus a straight CNT array. This point should be clarified.

Our reply: We thank the reviewer for his/her careful review and insightful comments.

The Q-factor of 20 was obtained by fitting the data from straight CNT array devices under excitations at 1111 cm^{-1} and 1639 cm^{-1}. Both frequencies lie outside the Reststrahlen band of hBN, meaning no coupling with hBN phonon polaritons. This value reflects the intrinsic Q-factor of the CNT array plasmons.

In contrast, the Q-factor of 60 was derived from the resonant peak's center frequency near 1600 cm^{-1}, which falls within the Reststrahlen band of hBN. Therefore, this value represents the Q-factor of the hybrid polaritons resulting from the coupling between the CNT array plasmons and hBN phonon polaritons. The intrinsically high Q-factor of hBN phonon polaritons (which experience zero electronic loss) contributes to the enhanced Q-factor observed in the hybrid system.

Furthermore, polaritons propagating within a nanocavity can exhibit further enhanced Q-factors. Previous literature has reported the Q-factor of hBN phonon polaritons in optical microcavities reaching values as high as 100~400 [*Nat. Commun. 8, 15624 (2017)*] [*Nat. Mater. 23, 499 – 505 (2024)*].

These factors collectively explain the observed enhancement of the Q-factor from 20 in straight devices to 60 in closed-loop CNT arrays.

2) Whispering gallery mode, WGM, scenario is still not fully clear based on the data itself. Could the authors potentially show a more direct visualization of the WGM, e.g., by placing a gold launcher at one location of the (larger) closed-loop array to visualize the propagating/circulating WGM? Another simple point to check is whether the WGM wavelength between 1563-1593 cm-1 in a resonance condition. Can the authors compare the round-trip distance around the cavity with

respect to the mode wavelength, and confirm if the resonance condition matches the experimental observations?

Our reply: We appreciate the reviewer's insightful comments and helpful suggestions.

Unfortunately, fabricating a gold launcher directly on the CNT array would introduce polymeric residues and contaminants that are nearly impossible to completely eliminate. Given the small size of the sample, these contaminations would significantly increase the loss of polaritons and severely compromise the acquisition of near-field optical signals. As a result, this approach is experimentally unfeasible.

To address the reviewer's question, we conducted additional numerical simulations, the results of which are presented in Figure R4. To align with the experimental conditions, we set the radial width of the CNT array equal to that of the experimental sample while intentionally extending the axial length to observe more oscillation periods. For clearer visualization of the polariton phase distribution, we positioned an electric dipole (indicated by the blue dot in Figure R4a) above the array to excite the polaritons and extracted the Re(Ez) component directly above the array.

The simulations clearly reveal the characteristics of whispering-gallery modes of the polaritons; as the incident frequency increases, the polariton wavelength decreases, resulting in more interference nodes within the standing wave pattern.

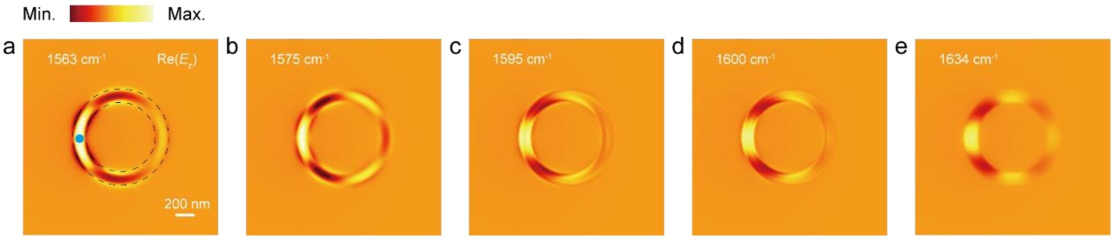

Figure R4. The evolution of whispering-gallery mode of polaritons in CNT-array/hBN at different frequencies. The excitation frequencies are 1563cm^{-1} (a), 1575cm^{-1} (b), 1595cm^{-1}(c), 1600 cm^{-1} (d) and 1634cm^{-1} (e), respectively. The blue dot in (a) represents the electric dipole for exciting the polaritons. The dashing black circles represents the boundary of the CNT array loop.

Furthermore, the direct fabrication of gold launchers without residual contamination on CNT arrays poses significant challenges, preventing the extraction of polariton phase information from experiments or the precise determination of their wavelengths for comparison. According to the polariton dispersion relation, the theoretical wavelength range is between 600 and 1000 nm, roughly matching the circumference of a single ring in the CNT array.

Additionally, previous studies have shown that when the cavity size is reduced to approximately $\sim\lambda_p$, polariton modes are dominated by standing waves confined along the cavity circumference [*Nat. Mater. 22, 838–843 (2023)*]. However, the quantitative conditions for low-order standing wave resonances remain challenging to analyze at this cavity size [*Nat. Commun. 8, 15624 (2017)*]. In conclusion, it remains uncertain whether the resonance conditions are strictly satisfied.

Therefore, we propose that CNT arrays support standing wave interference induced by whispering-gallery modes, which may manifest as either constructive or destructive interference. However, the specific resonance mode cannot yet be definitively identified from the available experimental data.

Response to Reviewer #4:

I co-reviewed this manuscript with one of the reviewers who provided the listed reports. This is part of the Nature Communications initiative to facilitate training in peer review and to provide appropriate recognition for Early Career Researchers who co-review manuscripts.

Our reply: We thank the reviewer for his/her appreciation of our work and for the positive evaluation.

Response to Reviewers' Comments (NCOMMS-25-86882B)

We sincerely thank all reviewers for their careful evaluation of our work and for their insightful and constructive feedback. Below are our detailed point-by-point responses.

Response to Reviewer #1:

The authors addressed all my comments, and the manuscript appears publishable without further revision.

Our reply: We sincerely thank the reviewer for his/her appreciation of our work and recommendation for the publication of our manuscript.

Response to Reviewer #3:

While the reviewer agrees that hybridization with hBN phonon polaritons can enhance Q-factor, the authors need to show quantitative evidence of Q-factor enhancement up to 60. Since high quality factor WGM is one of the central claims of the paper, emphasized both in abstract and main text, these points need to be clarified and the derivation and analysis of Q-factor needs to be more rigorously presented.

Besides, based on the additional simulations provided by the authors (Figure R2), the peak in their spectral evolution of near-field signal in a fixed on position within WGM cavity appears to be related to a geometric effect. Near field amplitude under the tip results from tip-launched polaritons under the tip interfering with those making a round-trip across the circumference of cavity. Changing frequency changes WGM wavelength, which shifts the phase of round-trip polaritons with respect to those launched under the tip. When frequency is continuously swept, this continuous increase in the relative phase shift will lead to destructive and to constructive and back to destructive interference, which looks like a peak in spectra. Q-factor derived from the FWHM of the standard fitting to this peak then does not (accurately) reflect the loss of WGM. Can the authors clarify this point?

Our reply: We appreciate the reviewer's insightful comments and helpful suggestions.

First, we wish to clarify the core novelty of our manuscript lies in the discovery of frequency-dependent topological polaritons arising from the coupling between hyperbolic plasmons and phonon polaritons in CNT-array/hBN heterostructures. In particular, we achieved a controllable topological transition of the polariton wavefront from hyperbolic to elliptical. The whispering-gallery mode (WGM) presented here serves as a natural extension and an in-depth investigation of this hybrid polariton mode, providing quantitative evidence of its low-loss characteristics and enhanced coherence

We agree with the reviewer's physical interpretation that the resonance peak of the WGM observed in our fixed-position near-field measurement may arise from the interference between the tip-launched field and the field that has completed one round trip along the cavity circumference. Consequently, the linewidth of such a peak should not be directly and exclusively interpreted as the intrinsic material loss.

In response, we wish to offer the following clarification:

1. The "Geometric Effect" is the Essence of Cavity Resonance:
The "geometric effect", i.e., the round-trip phase-matching condition requiring an integer multiple of 2π along the circumference, is precisely the fundamental physical mechanism underlying all optical microcavities, including whispering-gallery mode cavities and Fabry-Perot resonators. In this context, the resonance linewidth is governed by two factors: (a) the intrinsic propagation loss of the polaritons (including attenuation, radiation, and boundary scattering) and (b) the phase accumulation in the closed-loop geometry.

2. The Linewidth is a Valid Proxy for Loss:
In such interference-based resonant cavities, the contrast and sharpness of the resonance peak are inherently limited by the intrinsic propagation loss of the polaritons within the cavity.

If the propagation loss is high (short propagation length), the signal returning after one or multiple round trips is too weak to interfere significantly, resulting in a broad, low-contrast spectral fringe (corresponding to a low Q-factor). Conversely, if the propagation loss is extremely low, as achieved here through hybridization with low-loss hBN phonon polaritons, the polaritons survive for multiple round trips, enabling sharp constructive interference and a narrow spectral linewidth (corresponding to a high Q-factor).

Therefore, fitting the resonance peak to extract $\Delta\omega$ and calculating $Q = \frac{\omega_c}{\Delta\omega}$ is a standard and rigorous approach in nanophotonics and cavity physics to assess the effective Q-factor of a resonant mode. The extracted value of Q≈60 from our fitting indeed reflects the intrinsically low-loss nature and enhanced coherence of the closed-loop CNT/hBN hybrid polariton WGM resonance.

Revision in Supplementary Information:
To prevent over-interpretation, we have added the relevant section to explicitly define this value as the "effective Q-factor":
"We now explicitly describe the extracted value as the effective Q-factor under our specific near-field excitation and detection conditions, rather than the intrinsic material Q. We clarify that the linewidth is determined by both the round-trip attenuation and the phase-matching. Therefore, the Q-factor extracted from the FWHM provides an upper-bound estimate of the lifetime of the circulating hybrid polariton mode."

Response to Reviewer #4:

I co-reviewed this manuscript with one of the reviewers who provided the listed reports. This is part of the Nature Communications initiative to facilitate training in peer review and to provide appropriate recognition for Early Career Researchers who co-review manuscripts.

Our reply: We thank the referee for letting us know about his/her involvement in the Nature Communications initiative. It's commendable to see efforts towards training in peer review and recognizing the contributions of Early Career Researchers. The referee's commitment to this initiative is valuable for the research community.

The manuscript reports configurable plasmon–polaritons arising from the coupling between hyperbolic plasmons and phonon polaritons in CNT array/hBN heterostructures. Infrared (IR) nano-imaging is performed as a function of excitation laser energy, which reveals multiple branches of coupled phonon-plasmon modes. Topological transition of a hyperbolic isofrequency contour to an elliptical one is observed using IR nano-imaging and from numerical calculations. Plasmonic whispering-gallery modes are presented in closed-loop CNT array ring on a hBN substrate. Due to the lack of novelty and impact of the work, and multiple significant claims with insufficient evidence and premature data analysis, the reviewer does not recommend the publication of this manuscript in *Nature Communications*.

Comment 1: First and foremost, the main concern of the manuscript is the lack of novelty. Hyperbolic polaritons have been demonstrated numerous times in other vdW materials, with canonical examples including phonon polaritons hBN and α-MoO_3 as the authors have referenced as well as in nanopatterned hBN [Science 359, 892 (2018)]. Hyperbolic plasmons have also been demonstrated in multiple other vdW materials, including in ZrSiSe at infrared frequencies [Sci Adv 8, add6169 (2022)] and in $MoOCl_2$ at visible frequencies [Science 387, 786 (2025)]. The properties claimed by the authors (deep subwavelength confinement, broad spectral range, etc) are generic to these known (plasmonic) hyperbolic systems. Furthermore, the coupling-induced topological transition of polaritons has been repeatedly demonstrated in other vdW materials platforms, including in twisted α-MoO_3 [Nature Mater 19, 1307 (2020); Nature 582, 209 (2020)] or in graphene/α-MoO_3 [Nature Nanotech 17, 940 (2022); Nature Commun 13, 3719 (2022)]. Therefore, the reviewer does not find that the work provides a sufficient conceptual advance to warrant publication in *Nature Communications*.

Comment 2: Another significant concern is that the manuscript repeatedly makes claims that are not substantiated by sufficient experimental evidence or logical derivation, which compromises the scientific integrity of the manuscript.

2-i: The claim of "configurability" based on simple frequency dependence is misleading. This is a basic material property of CNT array/hBN device, but not an externally tunable feature that "configurability" typically implies. The authors' method of labelling "configurability" can be applied to most all vdW polaritonic materials. For example, phonon polaritons in hBN exist only within the Reststrahlen band. In this case, hBN phonon polaritons would also be "configurable" as one can switch phonon polaritons on and off simply by tuning frequency.

2-ii: The authors claim to be the "first" to demonstrate, "a whispering-gallery plasmonic mode confined in closed-loop CNT array on hBN." However, this claim of novelty is overstated. As the authors' own citations (Ref. 38) confirm, whispering-gallery plasmonic modes have already been demonstrated in other vdW materials, such as graphene. Therefore, the simple extension of this work to a CNT array does not seem to be a significant conceptual advance, and the manuscript fails to convey the importance of this observation in CNT arrays.

2-iii: In addition, the authors' claim of a whispering-gallery plasmonic mode itself is not convincing. Plasmonic whispering gallery modes are those that travel around a circumference of the cavity. Based on the main dataset shown in Fig. 4c, the reviewer does not see any clear signatures of plasmonic wave travel around the circular, donut shaped structure, forming a whispering-gallery mode. IR nano-imaging shown in Fig. 4c seems rather a result of tip-launched (or cavity edge launched) plasmons interacting with the entire cavity structure, forming a cavity-like nano-IR response, but not necessarily a whispering-gallery mode specific to the chiral motion of polaritons around the loop. More rigorous analysis needs to be performed to support their claim of whispering-gallery modes.

2-iv: The authors claim that "the synergy of the low-loss characteristics of the Luttinger liquid electronic state, uniform chirality, and near-atomic-level smooth, lossless ring-shaped interfaces enables efficient

optical field confinement and the formation of strong optical resonances," is highly speculative and lacks quantitative support. The manuscript provides no metrics to define, measure, and quantify the efficient optical field confinement and strong optical resonances. Furthermore, the authors do not demonstrate how their data supports the existence of or the connection to the low-loss Luttinger liquid, uniform chirality, or lossless interfaces. These claims are concerning because they are presented without any supporting evidence. At the very least, the authors should provide a quantitative comparison of the polariton quality factors in their straight and circular structures.

2-iv: The authors also make a direct comparison of their whispering gallery modes with that in Ref. 38, stating that "characteristic dimensions and performance have improved by nearly an order of magnitude" in their materials with "high compression ratio." However, this comparison to Ref. 38 is misleading. Dimensions may be smaller than the ones shown in Ref. 38, but it is unclear how the performance is improved. The authors' claim that their compression ratio is higher, but this is not supported quantitatively. The compression ratio (or perhaps the effective mode volume) of polaritons does not have one-to-one correspondence with the physical dimension of the cavity. While physical dimensions can be smaller, effective mode volume can be larger. One needs to perform a rigorous quantitative analysis to make a case for such a claim. Without a quantitative analysis, the authors' claim is unsubstantiated.

Other minor comments:

-hBN phonon polaritons are also hyperbolic. Can the authors comments on if the hyperbolicity of phonon polaritons play any role in the coupling and topological transition of hyperbolic plasmons in CNT array/hBN system?

-The authors selected an hBN flake with a thickness of 55 nm to demonstrate the polaritonic coupling. Why does this particular thickness allow the intensity of the hBN phonon-polaritons to match that of the CNT plasmons? Do the authors have any data or calculations to justify that 55 nm is the optimal thickness?
